# Improving Protein Expression, Stability, and Function with ProteinMPNN

**DOI:** 10.1021/jacs.3c10941

**Published:** 2024-01-09

**Authors:** Kiera H. Sumida, Reyes Núñez-Franco, Indrek Kalvet, Samuel J. Pellock, Basile I. M. Wicky, Lukas F. Milles, Justas Dauparas, Jue Wang, Yakov Kipnis, Noel Jameson, Alex Kang, Joshmyn De La Cruz, Banumathi Sankaran, Asim K. Bera, Gonzalo Jiménez-Osés, David Baker

**Affiliations:** † Department of Chemistry, University of Washington, Seattle, Washington 98195, United States; ‡ Institute for Protein Design, University of Washington, Seattle, Washington 98195, United States; § Center for Cooperative Research in Biosciences, Basque Research and Technology Alliance, Derio 48160, Spain; ∥ Department of Biochemistry, University of Washington, Seattle, Washington 98195, United States; ⊥ Howard Hughes Medical Institute, University of Washington, Seattle, Washington 98195, United States; # Berkeley Center for Structural Biology, Molecular Biophysics, and Integrated Bioimaging, Lawrence Berkeley Laboratory, Berkeley, California 94720, United States; ∇ Ikerbasque, Basque Foundation for Science, Bilbao 48013, Spain

## Abstract

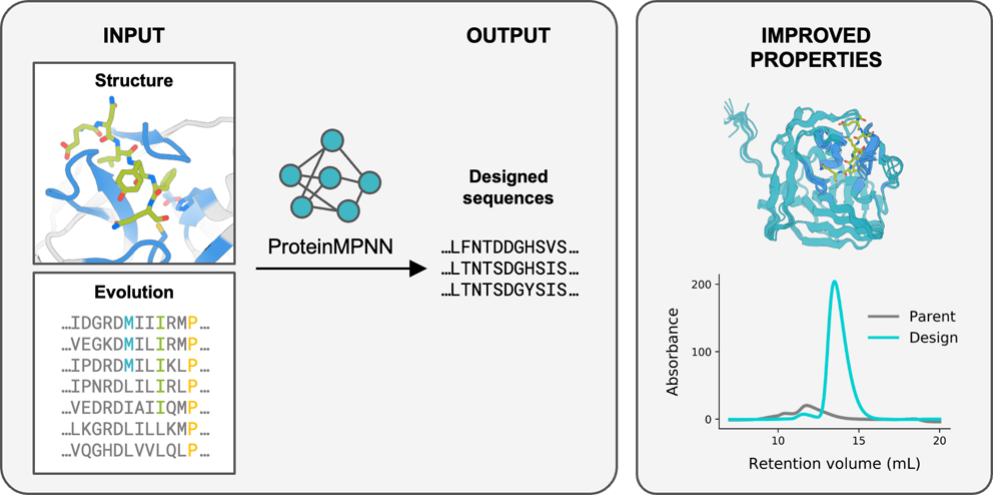

Natural proteins are highly optimized for function but are often difficult to produce at a scale suitable for biotechnological applications due to poor expression in heterologous systems, limited solubility, and sensitivity to temperature. Thus, a general method that improves the physical properties of native proteins while maintaining function could have wide utility for protein-based technologies. Here, we show that the deep neural network ProteinMPNN, together with evolutionary and structural information, provides a route to increasing protein expression, stability, and function. For both myoglobin and tobacco etch virus (TEV) protease, we generated designs with improved expression, elevated melting temperatures, and improved function. For TEV protease, we identified multiple designs with improved catalytic activity as compared to the parent sequence and previously reported TEV variants. Our approach should be broadly useful for improving the expression, stability, and function of biotechnologically important proteins.

## Introduction

Evolution has optimized function over stability in most natural proteins;^1^ as a result, they often exhibit poor solubility, thermostability, and expression in heterologous systems, all of which reduce the yield of functional protein.^2,3^ Many protein-based therapeutics and catalysts are limited in their industrial application by low stability, making protein stabilization a research area of increasing interest.^4,5^ Experimental methods such as directed evolution have been extensively used to optimize desirable features in proteins but are often prohibitively resource- and labor-intensive.^6,7^ Computational tools have been developed to achieve the benefits of directed evolution while minimizing experimental screening.^8−11^ PROSS (protein repair one-stop shop), for example, utilizes evolutionary information and Rosetta physics-based energy calculations to perform sequence redesign using a three-dimensional (3D) structure as input and has been shown to increase the soluble expression and thermostability of several natural proteins.^8^ More recently, advances in deep learning-based modeling of proteins have been applied to generate new variants of natural proteins, including language models that generate sequences for a given enzyme family or function,^11^ convolutional neural networks that leverage structural information for the prediction of gain-of-function mutations,^10^ and shallow neural networks for guiding combinatorial directed evolution.^12^

Deep learning-based tools for protein sequence design have shown success in the generation of novel proteins with excellent expression, solubility, and sub-angstrom accuracy to design models.^11,13,14^ ProteinMPNN generates highly stable sequences for designed backbones, and for native backbones, it generates sequences that are predicted to fold to the intended structures more confidently than their native sequences.^13^ We reasoned that ProteinMPNN could be applied to protein stability optimization and set out to develop a strategy for applying ProteinMPNN to natural proteins to increase solubility and stability. We chose as model systems one of the first proteins whose structure was solved, the oxygen storage protein myoglobin, and the widely used protease from tobacco etch virus (TEV).

## Results

### Protein Stabilization with ProteinMPNN

**Figure 1:**
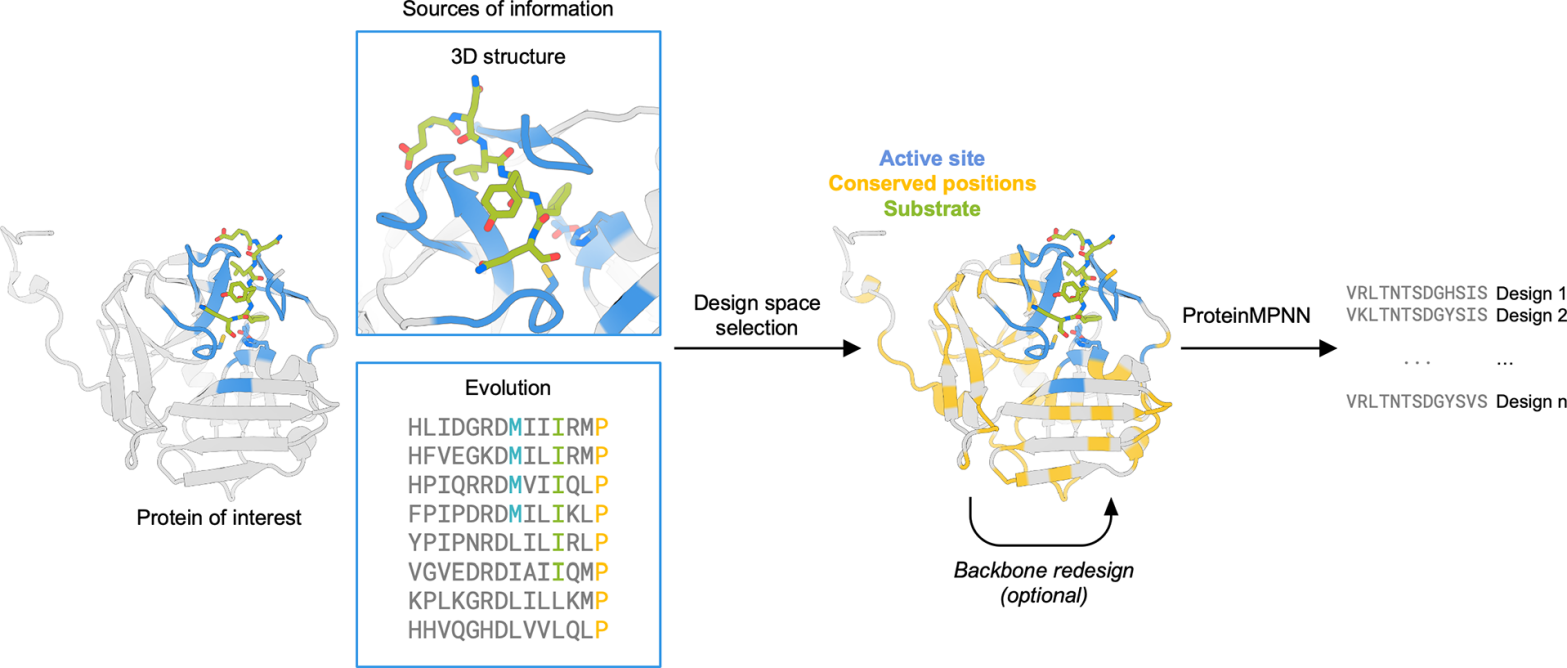
Design strategy for the optimization of protein expression and stability using ProteinMPNN. The design space is chosen to preserve the native protein function by fixing the amino acid identities of residues close to the ligand and those that are highly conserved in multiple sequence alignments. The protein backbone structure and fixed position information are input into ProteinMPNN, which generates new amino acid sequences likely to fold to the input structure. The backbone structure in loop regions can optionally be remodeled using RoseTTAfold joint inpainting to further idealize the input protein.

ProteinMPNN generates amino acid sequences that are predicted to fold into a given 3D structure. The method is purely structure-based and does not have access to functional information. Therefore, to retain protein function during sequence design, additional information must be provided to the network. We experimented with a range of approaches to retain functionality during the design process. In all targets, to preserve the catalytic machinery and substrate-binding site, we fixed the amino acid identities of the first shell functional positions—defined as those within 7 Å of the substrate in a ligand-bound crystal structure complex. For TEV protease, we used evolutionary information to further identify residues critical to activity. In myoglobin, we performed a limited backbone redesign to further stabilize the structure. With the design space selected, we generated sequences with ProteinMPNN, predicted the structures with AlphaFold2,^15^ and filtered by the predicted local distance difference test score (pLDDT) and Cα root-mean-square deviation (RMSD) to the input structure (Figure 1).

### Design of Myoglobin Variants with Increased Stability

We first applied our design strategy to the model protein myoglobin. Myoglobin binds heme to carry oxygen in mammalian muscle tissue,^16^ and has relevance in clinical applications as a biomarker,^17^ as a versatile platform for biocatalytic applications,^18−20^ and in food science as an ingredient in artificial meat products.^21−23^ Current efforts to create more stable variants of myoglobin have focused on the stabilization of the globin fold through stapling with cysteine-reactive noncanonical amino acids.^24,25^

**Figure 2:**
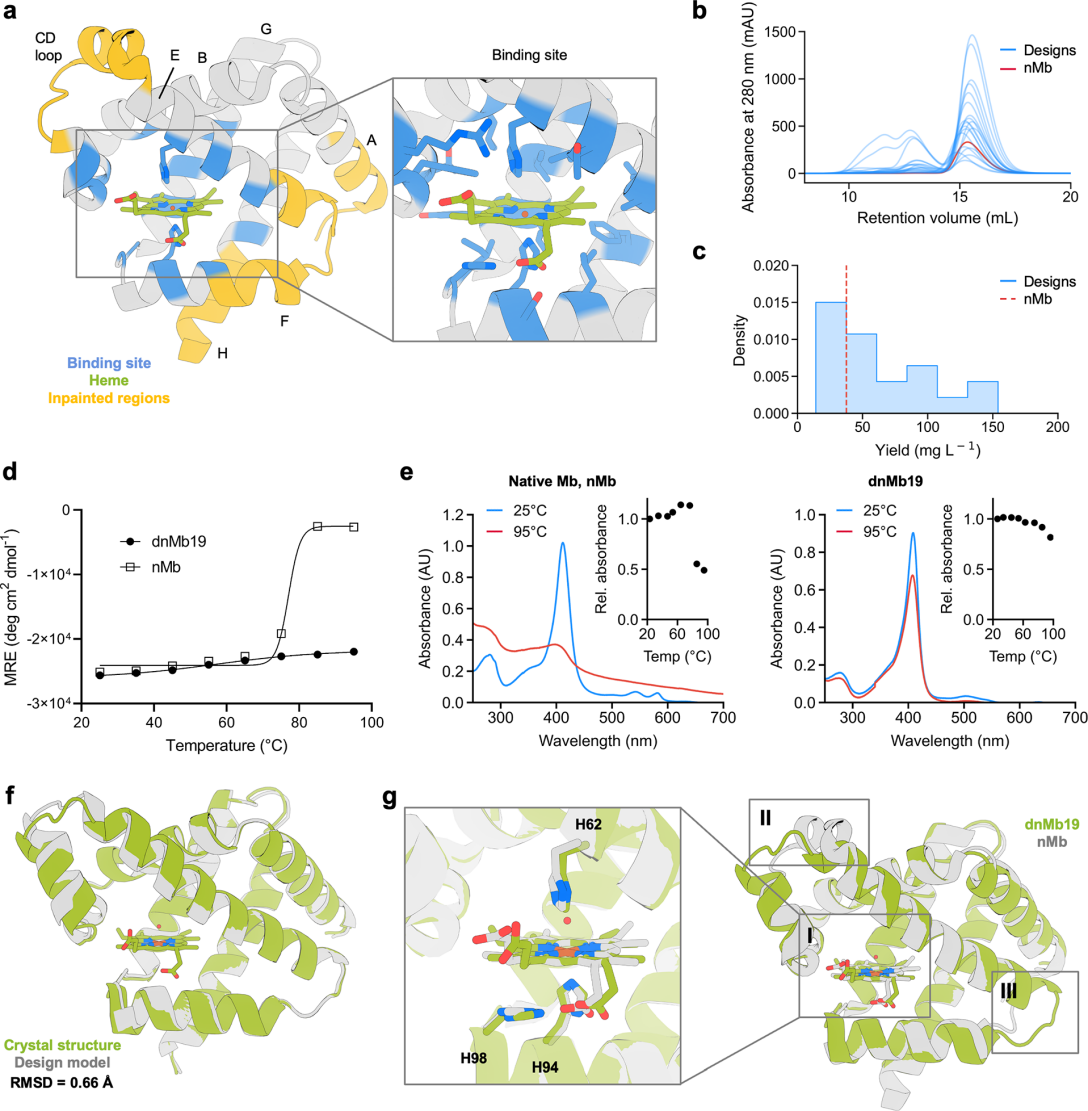
ProteinMPNN design improves myoglobin expression and thermostability. (a) Positions adjacent to the heme were kept fixed during the sequence design (shown in blue). Non-conserved regions (in yellow) were subjected to backbone remodeling. Inset shows the heme-binding site. (b) SEC traces of 20 designed myoglobin variants. (c) Soluble yield of myoglobin designs and native myoglobin (nMb, represented as a red dashed line). (d) CD melting temperature plots of dnMb19 compared to native myoglobin (signal reported in molar residue ellipticity (MRE)). (e) Absorbance plots of dnMb19 and native myoglobin (inset shows the temperature scan). (f) Structural alignment of the crystal structure (green) and AlphaFold2 (AF2) prediction (gray) of dnMb19. (g) Overlay of the crystal structure of native myoglobin (gray) and the crystal structure of dnMb19 (green, PDB: 8U5A). Non-conserved regions displayed in insets II and III were subjected to backbone redesign.

We applied the ProteinMPNN design protocol described above using a crystal structure of human myoglobin, nMb (PDB: 3RGK).^26^ To preserve the oxygen storage function, we fixed the identities of 17 positions located around the heme ligand in the heme-bound structure (Figure 2A). Sixty sequences were generated with ProteinMPNN and evaluated for their likelihood to recapitulate the myoglobin backbone coordinates using AlphaFold2 single-sequence predictions (see Supporting Information). Eight of the designs did so with high confidence (pLDDT > 85.0 and Cα RMSD < 1.0 Å; analogous single-sequence prediction of the native sequence yielded pLDDT = 50.6 and Cα RMSD = 7.5 Å). Four designs with close structural agreement in the heme-binding region were selected for experimental testing.

We also explored the limited backbone redesign of poorly ordered regions to attempt to further stabilize the protein. The globin superfamily, of which myoglobin is a member, has a fold made up of eight alpha helical regions, with diversity in the termini and two loop regions flanking the heme-binding pocket^27−29^ (Figure S1). We selected these less-conserved loop regions for backbone remodeling with RoseTTAFold joint inpainting (Figure 2A).^30^ We generated two distinct sets of designs with structural remodeling: one with the region joining helices E and F redesigned and one additionally including the CD-loop region (Figure 2A). From these remodeled backbones, we again performed sequence design with ProteinMPNN, with the heme-binding site kept fixed as described above. Following filtering on structure prediction metrics (Figure S2), an additional 16 sequences were selected for experimental testing. All 20 tested myoglobin designs have 41–55% sequence identity with the most similar protein (a myoglobin in all cases) in the UniRef100 database^31^ (Table S1).

Synthetic genes encoding the designs and the parent sequence, nMb, were expressed in *E. coli*. The heme-loaded *holo*-proteins were purified via immobilized metal affinity chromatography (IMAC) and size exclusion chromatography (SEC). All designs were expressed and were monomeric by SEC (Figure 2B). Thirteen of the twenty designs had higher levels (up to a 4.1-fold increase) of total soluble protein yield compared to that of native myoglobin (Figure 2C). All 20 designs had similar heme-binding spectra to native myoglobin, with agreement in the Soret maximum (407–413 nm vs 409 nm in native) and Q-band features (500, 537, 582, and 630 nm), suggesting the preservation of the native heme-binding mechanism (Figure S3).

The thermal stabilities of eight highly-expressing designs (six and two designed with and without backbone remodeling, respectively) were evaluated by circular dichroism (CD) spectroscopy. All eight designs had higher melting temperatures than that of native myoglobin, with six remaining fully folded at 95 °C (native myoglobin melts at 80 °C; Figures 2D and S4). Heme binding was also evaluated over a temperature gradient to determine the functional thermal stability. All designs preserved heme binding at higher temperatures than native myoglobin (as monitored by changes to the Soret band wavelength and intensity in the UV/vis spectrum), with five designs maintaining significant heme-binding at 95 °C (Figure S5). One of the five designs, dnMb19, generated with the more aggressive backbone remodeling strategy, showed a much higher thermal stability of heme binding compared to native myoglobin (Figure 2E). Overall, remodeling regions of the myoglobin backbone with inpainting increased the success rate for retaining heme-binding at elevated temperatures.

To understand the structural basis of these improvements in stability, we determined the crystal structure of dnMb19 (2.0 Å resolution, PDB: 8U5A). We found that it closely agreed with the design model (0.66 Å Cα RMSD, Figure 2F), including the regions remodeled with inpainting. Native side chain contacts with the heme group are largely preserved in dnMb19 (Figure 2G, inset I). Outside of the heme-binding site, the crystal structure confirms the structural changes introduced by inpainting: the C and E helices were elongated as designed and connected by a new loop (Figure 2G, inset II); the loop connecting the E and F helices has a new conformation, and the F helix was straightened through the replacement of PRO88 with GLU89 (Figure 2G, inset III). The Cα RMSD over the inpainted regions between the crystal structure and the design model is 0.88 Å, with the largest deviation being in the CD-loop region (1.51 Å). These results illustrate the power of RoseTTAFold joint inpainting and ProteinMPNN to accurately remodel native protein backbones while increasing solubility, thermostability, and functional stability.

### Design of TEV Protease Variants with Improved Stability and Catalytic Activity

To explore the utility of ProteinMPNN sequence design for stabilizing enzymes, we next applied our design strategy to the cysteine protease from tobacco etch virus (TEV). TEV protease is widely used in biotechnological applications to specifically cleave between glutamine and serine in its recognition sequence (ENLYFQ/S) to remove purification tags from recombinant proteins. However, TEV protease has suboptimal properties, including low soluble yield from heterologous expression, low thermostability, and poor catalytic activity. These properties often necessitate long incubation times and result in incomplete cleavage.^32^

**Figure 3:**
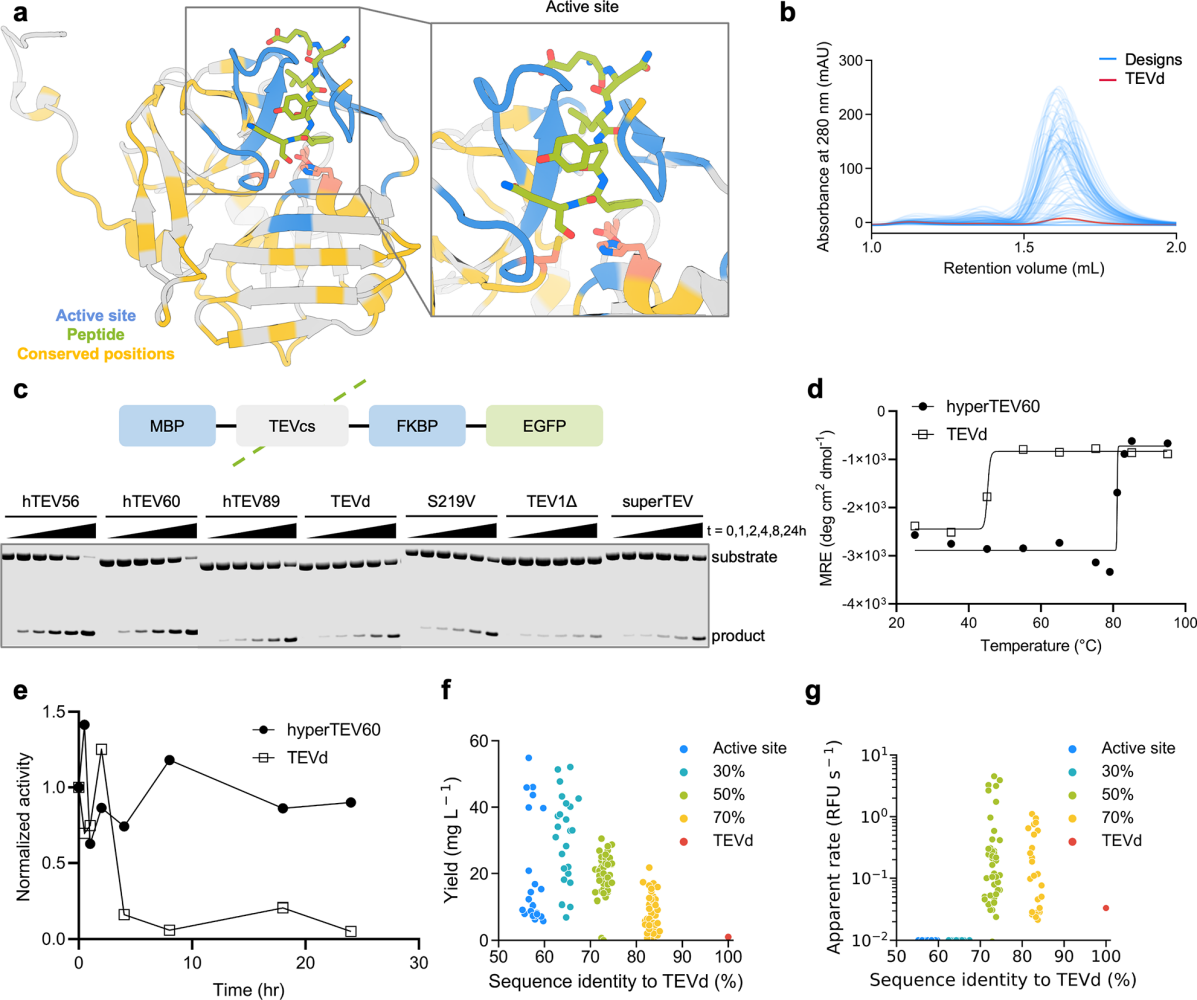
ProteinMPNN sequence design improves TEV protease expression, thermostability, and catalytic efficiency. (a) TEVd (PDB: 1LVM) input structure with positions fixed during redesign highlighted. Active site residues surrounding the substrate (blue), 50% most highly conserved residues (yellow), and catalytic residues (pink) are highlighted. Inset shows a zoomed-in view of the active site region. (b) SEC traces of the designed TEV variants. (c) Diagram of TEV substrate (top) and fluorescent gel image of TEV cleavage reactions at various time points (bottom). (d) CD melting temperature plots of the designed and native TEV (signal reported in molar residue ellipticity (MRE)). (e) Benchtop stability comparison of native TEVd and the designed variant assessed as activity measured over time incubated at 30 °C before inclusion in the assay. (f) Decreased evolutionary constraints correlate with higher soluble expression levels. Legend indicates regions fixed during the design (all designs have the active site fixed). (g) Designs made with the active site and 50% most conserved residues fixed during design exhibited the highest catalytic activity. Raw apparent rate is reported in relative fluorescence units (RFU) per second.

We applied our sequence design strategy to TEV protease starting from an autolysis-resistant S219D variant, TEVd (PDB: 1LVM).^33^ We defined the active site residues as described above to be fixed during redesign. We additionally fixed the amino acid identities of residues that are most conserved within the protein family (determined from a sequence alignment generated against UniRef30^31^), as residues distant from the active site can contribute significantly to function.^34^ We ranked each amino acid identity at each position by the degree of conservation in the sequence alignment and varied the percentage of these most highly conserved residues to fix during sequence redesign between 30 and 70%. We generated four distinct sets of designs that fixed the amino acid identities of just the active site residues or the active site residues and 30, 50, and 70% of the most conserved residues in the TEV family (Figure 3A, see Supporting Information). A total of 144 sequences were generated with ProteinMPNN, which were all predicted with high confidence to fold to the TEV structure by AlphaFold2 (pLDDT > 87.5; native TEV is predicted with pLDDT = 90) and possess 55 to 85% sequence identity to the parent sequence. All 144 designs were selected for experimental testing.

Synthetic genes encoding the designs, the parent sequence, TEVd, and several previously reported TEV variants were expressed in *E. coli*, and the resultant proteins were purified via IMAC and SEC. 134 of 144 designs solubly expressed and eluted as monomers by SEC (Figure 3B). 129 of 144 designs exhibited higher levels of soluble expression than TEVd (TEVd average yield = 1 mg/L culture, design average yield = 20.1 mg/L culture (Figure 3F)).

We evaluated catalytic activity using a previously described^7^ coumarin derivative with 7-amino-4-trifluoromethylcoumarin conjugated to the C-terminus of the TEV substrate peptide Ac-ENLYFQ (Figure S7A). Purified protein was incubated with the peptide-coumarin substrate, and 64 designs displayed progress curves with fluorescence above the background, indicating substrate turnover (Figure S7B and S7C). Designs made with no evolutionary constraints had improved soluble expression over the parent but were not active on the peptide substrate, while designs with the highest activities were designed with the top 50% most conserved residues fixed (Figure 3F,G). We performed detailed kinetic analysis of three highly active designs from the 50% design method—hyperTEV56, hyperTEV60, and hyperTEV89—and the parent sequence TEVd.^8^ The designs displayed improved catalytic efficiencies (*k*_cat_/*K*_m_) compared to TEVd, with up to 26-fold improvements (Table 1 and Figure S8).

**Table 1:** Kinetic Parameters for TEV Redesigns and the Parent TEV Variant

variant	*k*_cat_ (min^–1^)	*K*_m_ (μM)	*k*_cat_/*K*_m_ (μM^–1^ min^–1^)	fold improvement in *k*_cat_/*K*_m_ over parent
hyperTEV56	0.0106 ± 0.0005	1.4 ± 0.2	0.0077	20
hyperTEV60	0.014 ± 0.002	1.4 ± 0.4	0.01	26
hyperTEV89	0.0050 ± 0.0001	2 ± 1	0.0024	6.2
TEVd	0.0023 ± 0.0003	6 ± 3	0.00039	

Next, we tested the most active designs with a fusion protein substrate to assess their performance on the target application of tag removal. The designs and a set of previously engineered TEV proteases^32,33,35−37^ were incubated at 30 °C with the fusion protein substrate MBP-TEVcs-FKBP-EGFP, where MBP is the maltose-binding protein, TEVcs is the TEV peptide cleavage site (ENLYFQS), FKBP is the FK506-binding protein, and EGFP is an enhanced green fluorescent protein. The extent of proteolysis was evaluated by monitoring the accumulation of the cleaved product via sodium dodecyl sulfate-polyacrylamide gel electrophoresis (SDS-PAGE) (Figure S9). Two designs, hyperTEV56 and hyperTEV60, exhibited significantly higher rates of cleavage of protein substrate compared to the parent TEVd, yielding 50% cleaved product at ∼4 h of incubation, while TEVd required 24 h to reach an equivalent yield. The designs also outperformed other published TEV variants, with 30% turnover for superTEV, 15% turnover for TEV1Δ, and 50% turnover for S219V at 24 h of incubation (Figures 3C and S10A). Straight-line fits of product accumulation and substrate depletion reveal catalytic efficiencies that corroborate those determined in the peptide assay (Figure S10B). In the peptide assay, the gains in catalytic efficiency are primarily due to increases in *k*_cat_, which could reflect a higher fraction of enzyme in a catalytically competent state (see below).

Analysis by CD spectroscopy of TEVd and the most active design, hyperTEV60, indicated an approximate melting temperature of 84 °C for hyperTEV60, 40 °C higher than that of TEVd (Figures 3D and S11), and to the best of our knowledge, higher than that of any previously described TEV variant. To further probe the stability of the designed variant, TEVd and hyperTEV60 were incubated at 30 °C for various times and then used in the peptide–coumarin cleavage assay. After 4 h of incubation, hyperTEV60 retained 90% of its original cleavage activity, while TEVd was reduced to 15% of its original activity (Figure 3E), indicating a significant improvement in benchtop stability.

Given that the catalytic and substrate-binding residues were kept fixed during the design with ProteinMPNN, it is notable that significant improvements in *k*_cat_ were observed with both the peptide and protein substrates. Mutations distal to the active site can influence catalytic activity through the stabilization of catalytically productive conformational states^38,39^ or global conformational changes.^40^ To investigate if the stabilization of functional conformational states may be involved in activity enhancement, we performed microsecond molecular dynamics (MD) simulations on TEV–peptide complexes to probe the impact of the introduced mutations on the overall protein dynamics. A general rigidification of loop regions distributed across the structure was observed in designs compared to TEVd (Figure S12A). This backbone rigidification in distal regions not directly involved in substrate binding may be related to allosteric improvement of substrate binding, as reflected by the two- to threefold lower *K*_m_ values measured for the designed variants (Table 1). Rigidification in the region spanning residues 115 to 124 appeared to correlate with activity; the highest activity design, hyperTEV60, was most rigid, while TEVd and a design with no activity on the peptide substrate were the most flexible in this region (Figure S12B). These trends were also observed in the per-residue pLDDT analysis of AlphaFold2 ensemble predictions (Figure S12C). In all designs, we observed a decrease in the population of catalytically competent conformations of the Cys-His dyad (*d*_N-SH_) compared to TEVd, but this shift was least significant in hyperTEV60, in agreement with its higher relative *k*_cat_ (Figure S13). These notable differences may begin to explain how ProteinMPNN enables substantial activity enhancements without explicit design elements to improve function. It is also possible that the major contribution to the increase in *k*_cat_ is from an increase in the fraction of the protein in the catalytically competent state more globally.

## Discussion

We show that the expression, stability, and function of native proteins can be improved using ProteinMPNN, guided by the available sequence and structural information. For both TEV protease and human myoglobin, multiple variants were identified that showed higher soluble yield and thermostability than the native protein. The best of the TEV protease designs have higher apparent catalytic efficiency on peptide and protein substrates than the parent enzyme and previously reported variants. While the optimal number of residues to maintain (and perhaps enhance) function may have to be determined empirically for each case, the simplicity of our procedure and the computational efficiency and ease of use of ProteinMPNN make this straightforward, and the number of variants that need to be tested is far smaller than that in typical experimental screens. We expect that our approach will be widely useful for improving the expression, stability, and function of biotechnologically important proteins.

## Supporting Information Available

Computational and experimental methods, details of crystallographic data collection, sequences of designs, structural diversity of natural and designed myoglobin variants; *in silico* metrics of myoglobin designs grouped by a method; raw thermostability, spectrophotometry, and activity data; raw data from Michaelis Menten fitting of TEV activity assays; data from MD simulations; sequence similarity analysis of myoglobin designs; and mass spectrometry data for purified myoglobin designs (PDF)
Computational models of designed proteins (ZIP)
