# Supplementary material for: Improving Protein Expression, Stability, and Function with ProteinMPNN
Source: J Am Chem Soc. 2024 Jan 9;146(3):2054–61. doi: 10.1021/jacs.3c10941 (PMC10811672; doi:10.1021/jacs.3c10941)
Supplement: Supplementary file 1 — ja3c10941_si_001.pdf [file ja3c10941_si_001.pdf]

Supplementary information

Improving protein expression, stability, and function with ProteinMPNN

Kiera H. Sumida[1,2], Reyes Núñez-Franco[3], Indrek Kalvet[2,4,5], Samuel J. Pellock[2,4], Basile I. M. Wicky[2,4], Lukas F. Milles[2,4], Justas Dauparas[2,4], Jue Wang[2,4], Yakov Kipnis[2,4,5], Noel Jameson[1], Alex Kang[2], Joshmyn De La Cruz[2], Banumathi Sankaran[6], Asim K. Bera[2,4], Gonzalo Jiménez-Osés[3,7], David Baker[2,4,5]*

[1]Department of Chemistry, University of Washington, Seattle, WA, USA.

[2]Institute for Protein Design, University of Washington, Seattle, WA, USA.

[3]Center for Cooperative Research in Biosciences, Basque Research and Technology Alliance, Derio, Spain.

[4]Department of Biochemistry, University of Washington, Seattle, WA, USA.

[5]Howard Hughes Medical Institute, University of Washington, Seattle, WA, USA.

[6]Berkeley Center for Structural Biology, Molecular Biophysics, and Integrated Bioimaging, Lawrence Berkeley Laboratory, Berkeley, CA, USA.

[7]Ikerbasque, Basque Foundation for Science, 48013 Bilbao, Spain.

* e-mail: dabaker@uw.edu

Content

Experimental methods

Computation Details 2

Supplementary Figures 5

Crystallographic Data 7

Sequence Information 21

References 24

33

EXPERIMENTAL METHODS

ProteinMPNN design of myoglobin. For fixed-backbone sequence redesign, the crystal structure of human myoglobin (PDB: 3RGK)[1] was used as input to ProteinMPNN, and 17 positions located around the heme were excluded from design. Three temperatures (0.1, 0.2, and 0.3) were sampled, with 20 sequences generated per temperature. Cysteine and methionine were excluded from the amino acid identities that could be installed during design. A model of ProteinMPNN trained with 0.2 Å noise applied to training set protein backbones was used to perform sequence generation. For combined sequence and backbone redesign, two strategies were employed. First, the sequence and structure from the crystal structure of human myoglobin (PDB: 3RGK) were input to RF_{joint}, with the N- and C-termini and loop region between helices 5 and 6 masked, to generate new secondary structure in these regions (RoseTTAFold joint inpainting). Ten backbones were generated with this strategy. In a more aggressive strategy, helix 4 and its adjoining loops, as well as both termini and the loop joining helices 5 and 6, were masked. Twenty backbones were generated with this strategy. Following backbone redesign, 60 sequences were generated per backbone with ProteinMPNN, keeping heme-binding positions fixed as described above. Comparison of the structural diversity introduced with RF_{joint} inpainting and the native globins is presented in Figure S1.

Sequences generated with ProteinMPNN were predicted with AlphaFold2, using model 4 with 10 recycling steps. Structural templating with MSAs was not used for prediction. Designs with only sequence redesign were filtered to Cα RMSD < 1.0 Å and pLDDT > 85.0. Designs with sequence redesign and backbone redesign on the termini and the loop connecting helices 5 and 6 were filtered to Cα RMSD < 0.8 Å and pLDDT > 90.0. Designs with sequence redesign and backbone redesign on the termini, helix 4, and the loop connecting helices 5 and 6 were filtered to Cα RMSD < 0.6 Å and pLDDT > 90.0 (see Figure S2 for details). Predicted models passing these criteria were finally evaluated by eye and those recapitulating finer structural details of the heme binding pocket (low backbone deviation after global alignment to the structure of 3RGK; close agreement with the placement of heme-coordinating histidine side chain) were selected for experimental testing. Four designs generated with only sequence design, and 16 designs with sequence and backbone design were selected for experimental testing (10 with both loops remodeled, and 6 with one loop remodeled).

Fixed residue selection for TEV protease. Active site positions were defined as residues containing backbone atoms within 7 Å of the substrate or sidechain atoms within 6 Å of the substrate in the ligand-bound crystal structure of autolysis resistant TEV variant S219D (PDB: 1LVM)[2]. Highly conserved residues, determined with a multiple sequence alignment (MSA), were also fixed during sequence redesign. To generate the MSA, four iterative HHblits searches[3] were performed against the UniRef30 database (accessed June 30, 2020) at E-value cutoffs of 1e-50, 1e-30, 1e-10, and 1e-4, and the final result was filtered for 90% identity redundancy, 50% coverage, and 30% minimum query identity. Within the sequence alignment, we identified the frequency of each amino acid at each position and found the most highly conserved amino acid identity at each position. We then ranked each position by how highly conserved the most frequent amino acid identity was, and selected the top 30%, 50%, and 70% most conserved positions to fix during sequence design.

ProteinMPNN design of TEV protease. The crystal structure of TEVd (PDB: 1LVM) was used as structural input to ProteinMPNN, and active site and conserved residues were excluded from design. Cysteine was excluded from the amino acid identities that could be installed during design. Three temperatures (0.1, 0.2, and 0.3) were sampled during design. A model of ProteinMPNN trained with 0.2 Å noise applied to training set protein backbones was used to perform sequence generation. 24 sequences were generated with only the active site residues fixed, 24 sequences were generated with the active site and the 30% most highly conserved positions fixed, 48 sequences were generated with the active site and the 50% most highly conserved positions fixed, and 48 sequences were generated with the active site and the 70% most highly conserved positions fixed.

Sequences generated with ProteinMPNN were predicted with AlphaFold2, using model 3 with 6 recycling steps. Both designs and native TEV predicted with low confidence if given only the single sequence and minimal recycling steps; we found that structural templating with MSAs was necessary for accurate prediction. To generate MSAs of each design for structure prediction, the MSA of the parent sequence was used, and the parent sequence was swapped for the design

sequence. All sequences generated were predicted with Cα RMSD < 2.0 Å and pLDDT > 85.0 and were predicted to maintain critical structural features in the active site. Thus, all were ordered for experimental characterization.

Expression and purification of myoglobin designs. Double-stranded DNA fragments encoding the designs (codon-optimized for bacterial expression) were purchased from Integrated DNA Technologies (IDT) as eBlocks™ Gene Fragments. Following the Golden Gate cloning protocol,[4] the DNA fragments encoding design sequences and including overhangs suitable for a *Bsa*I restriction digest were cloned into a custom pET29b(+) target vector containing lethal ccdb gene, and C-terminal SNAC[5] and hexahistidine tags (#191551, Addgene). This yielded final expressed sequences as: MSG<design>GSGSHHWGSTHHHHHH. Assembled plasmids containing the designs were transformed into *E. coli* BL21(DE3) by heat shock. DNA was incubated on ice with competent cells for 30 minutes, followed by 30 second heat shock at 42 °C, and 2 minute incubation on ice. 100 μL rich medium (super optimal broth with catabolite repression) was added to transformed cells and samples were incubated at 37 °C, 1050 r.p.m. on a Heidolph shaker for 1 hour. The cells were subsequently spread on LB-agar plates containing 100 μg/mL kanamycin and incubated at 37 °C under 220 r.p.m. shaking for 18 hours. Single colonies were picked, and the DNA fragments encoding the designs were amplified following a colonyPCR protocol using GoTaq® Green DNA polymerase master mix (#M7122; Promega) and T7 reverse and forward primers. The PCR products identified to contain DNA of appropriate size (~600 bp) based on agarose gel (1.2%) electrophoresis with SybrSafe dye were sent to Sanger sequencing (GeneWiz/Azenta) for sequence-verification. Single colonies containing the correct design sequences were grown up in 5 mL TB-II media containing 50 μg/mL kanamycin, over 16 hours at 37 °C. 2 mL of the grown culture was used to inoculate 40 mL TB-II media containing 50 μg/mL kanamycin and the rest used for plasmid extraction following the Qiagen QIAprep MiniPrep protocol. The 40 mL cultures were grown at 37 °C for 4 hours, after which protein expression was induced with the addition of 1 mM IPTG, and the cultures were incubated at 18 °C for 20 hours. Pellets were harvested by centrifugation at 4,198 g for 8 minutes and resuspended in a lysis buffer containing 25 mM Tris-HCl, 300 mM NaCl, 25 mM imidazole, 0.01 mg/mL DNAse, 0.1 mg/mL lysozyme, and a Pierce protease inhibitor tablet. 200 μM hemin (from 12 mM stock in 0.5 M aq. NaOH) was added to the resuspended cells. Lysis was performed by ultrasonication (13 mm probe, 2.5 mins, 10s on, 10s off, 65% amplitude). Lysate was collected by centrifugation at 15,000 xg for 20 minutes and applied to Ni-NTA resin that was equilibrated with wash buffer (25 mM Tris-HCl, 300 mM NaCl, 25 mM imidazole, pH 8.2). The resin was washed with 50 column volumes (CV) of wash buffer. Protein was eluted with 1.2 CV of elution buffer (25 mM Tris-HCl, 300 mM NaCl, 300 mM imidazole, pH 8.2) and further purified via size exclusion chromatography (SEC) using a Superdex Increase 75 10/300 GL column (GE Healthcare) on ÄKTAxpress (GE Healthcare) instrument at 0.8 mL min-1 flow rate. The monomeric or smallest oligomeric fractions of each run (eluting at approximately 15 ml) were collected. The obtained chromatograms are presented in Figure S6.

Yields of purified hemoproteins were determined based on the absorbance of the Soret maximum (407-413 nm). The corresponding extinction coefficients were measured for each protein using the hemochromagen assay, according to the method of Berry and Trumpower.[6] A reported extinction coefficient of 188 $mM^{-1}\cdot cm^{-1}$ was used for native myoglobin.[7]

Expression and purification of TEV designs. Double-stranded DNA fragments encoding the designs (codon-optimized for bacterial expression) were purchased from Integrated DNA Technologies (IDT) as eBlocks™ Gene Fragments. Following the Golden Gate cloning protocol,[4] the DNA fragments encoding design sequences and including overhangs suitable for a *Bsa*I restriction digest were cloned into a custom pET29b(+) target vector containing lethal ccdb gene, and C-terminal SNAC[5] and hexahistidine tags (#191551, Addgene). This yielded final expressed sequences as: MSHHHHHHSG<design>GS. Vectors containing TEV designs were transformed into *E. coli* BL21(DE3) (New England BioLabs) by heat shock. DNA was incubated on ice with competent cells for 30 minutes, followed by 10 second heat shock at 42 °C, and 2 minute incubation on ice. 100 μL rich medium (super optimal broth with catabolite repression) was added to transformed cells and samples were incubated at 37 °C, 1050 rpm on a Heidolph shaker for 1 hour. Entire transformations were transferred to 900 μL of TBM-5052 autoinduction expression medium containing 50 μg/mL Kanamycin. Expression cultures were incubated at 37 °C, 1050 rpm for 20 hours. Pellets were harvested by centrifugation at 4,000 g for 10 minutes and lysed with BPER lysis reagent containing 6.25 Units/mL benzonase (4 uL / 40 mL at 250 U/μL), 0.1 mg/mL lysozyme, and 1 mM PMSF. Lysate was collected by centrifugation at 4,000 xg for 20 minutes and applied to Ni-NTA resin that was equilibrated with wash buffer (20 mM Tris-HCl, 300 mM NaCl, 25 mM imidazole, pH

8.0). The resin was washed with 25 column volumes (CV) of wash buffer. Protein was eluted with 250 μL of elution buffer (20 mM Tris-HCl, 300 mM NaCl, 540 mM imidazole, pH 8.0) and further purified via size exclusion chromatography (SEC) in an S75 5/150 GL increase column (GE Healthcare). Protein collected from SEC was normalized to 1 μM where possible.

In scale-up experiments, constructs in competent cells were plated on LB-agar plates containing 100 μg/mL kanamycin and individual colonies were picked and Sanger sequenced to verify gene sequences. 50-mL cultures of TBM-5052 autoinduction media with 50 μg/mL Kanamycin were inoculated with a scrape of sequence-verified transformed competent cells from glycerol stock and grown at 37 °C, 200 rpm for 20 hours. Cells were harvested by centrifugation at 10,000 xg for 10 minutes, resuspended in 30 mL of wash buffer (20 mM Tris-HCl, 300 mM NaCl, 25 mM imidazole, pH 8.0) containing 0.01 mg/mL DNase, 0.1 mg/mL lysozyme, and a protease inhibitor tablet (Thermo Scientific Pierce), and lysed by sonication. Lysate was collected via centrifugation at 18,000 xg for 40 minutes and applied to Ni-NTA resin that was equilibrated with wash buffer. The resin was washed with 30 CV of wash buffer. Protein was eluted with 4 mL of elution buffer and concentrated to 1 mL in a 3K protein concentrator (Millipore Sigma). Concentrated protein was purified by SEC as described above.

Expression and purification of MBP-TEVcs-FKBP-EGFP construct. The protease substrate FKBP-EGFP was cloned into an *E. coli* expression vector containing an N-terminal maltose binding protein (MBP), a TEV protease recognition site, and a C-terminal His-6 tag. The FKBP-EGFP coding sequence was obtained from Addgene #106924, with a 4X GGS linker between FKBP and EGFP. Vector containing the protease substrate was transformed into *E. coli* BL21(DE3) by heat shock. Cells were transferred to 4 0.5-L LB medium cultures with 10 μg/mL Carbenicillin and 10 μg/mL Chloramphenicol and incubated at 37 °C, 200 rpm until optical density reached 0.5 AU, at which point expression was induced with 1 mM IPTG. Temperature was reduced to 18 °C and cells were incubated for an additional 18 hours. Cells were harvested by centrifugation at 10,000 xg for 10 minutes, resuspended in 30 mL of wash buffer (20 mM Tris-HCl, 300 mM NaCl, 25 mM imidazole, pH 8.0) containing 0.01 mg/mL DNase, 0.1 mg/mL lysozyme, and a protease inhibitor tablet (Thermo Scientific Pierce), and lysed by sonication. Lysate was collected via centrifugation at 18,000 xg for 40 minutes and applied to Ni-NTA resin that was equilibrated with wash buffer. The resin was washed with 30 CV of wash buffer. Protein was eluted with elution buffer until resin no longer appeared yellow and concentrated to 1 mL in a 3K protein concentrator (Millipore Sigma). Concentrated protein was purified by SEC as described above.

Kinetic characterization of designed proteases. Designs were initially screened for activity on a peptide-coumarin conjugate substrate (WuXi) of the TEV recognition sequence (ENLYFQ) fused to a fluorescent coumarin derivative, 7-amino-4-trifluoromethylcoumarin. The N-terminus of the peptide bears an acetyl modification and the C-terminus is conjugated to the coumarin group via an amide bond. Initial activity screen was performed in 50 mM Tris-HCl, 50 mM NaCl, pH 8.0 buffer containing freshly prepared 2 mM DTT. Reactions contained 500 nM protein and 10 μM substrate at a total volume of 30 μL. Protein and substrate were rapidly mixed and monitored for fluorescence at excitation 400 nm, emission 492 nm at room temperature (RT) for 5 hours in a BioTek Synergy Neo2 microplate reader.

For detailed kinetic characterization, reactions were performed in 50 mM Tris-HCl pH 8.0 containing 50 mM NaCl, 1 mM EDTA, and freshly prepared 2 mM DTT. For TEV redesigns, reactions contained 50 nM protein and substrate concentration ranging from 0.1 μM to 10 μM at a total volume of 30 μL. Protein and substrate were rapidly mixed and monitored for fluorescence at excitation 400 nm, emission 492 nm at RT for 2 hours in a BioTek Synergy Neo2 microplate reader. Fluorescent signal was converted to concentration of cleaved coumarin product using a calibration curve of 7-amino-4-trifluoromethylcoumarin. Reactions were performed in triplicate and each technical replicate was separately fitted to a Michaelis Menten model. Expressed uncertainty in k_{cat} and K_m is the standard deviation between technical replicates.

Screening of designed proteases on fusion protein MBP-TEVcs-FKBP-EGFP. Reactions were performed in 50 mM Tris-HCl, 50 mM NaCl, 1mM EDTA, pH 8.0 buffer containing freshly prepared 2 mM DTT. Reactions contained 60 nM protein and substrate concentrations ranging from 2 μM to 17 μM. Reactions were incubated at 30 °C and at 0, 1, 2, 4, 8, and 24 hours, 10 μL aliquots were quenched in 10 μL of 2X Laemmli loading buffer and subsequently frozen in liquid nitrogen. Samples were analyzed by SDS-PAGE and imaged for EGFP fluorescence at 488 nm on a LI-COR Odyssey M

imager. Band intensities were quantified with ImageJ software and converted to concentration using a standard curve prepared of known amounts of cleaved substrate with fluorescence gel imaging. A straight-line fit was applied to the initial velocities using GraphPad Prism. Points represent the averages of 3 technical replicates and error bars represent the standard deviations.

Benchtop stability characterization of TEV redesigns. Samples of purified enzyme were incubated at 30 °C for 0.5, 1, 2, 4, 8, 18, or 24 hours before being used in the previously described peptide-coumarin cleavage assay. Activity of samples was defined as initial rate of turnover and normalized to initial rate at incubation of t = 0 hrs.

Spectrophotometric measurements. UV-Vis spectra of purified holo-proteins (myoglobin variants) in the 230-800 nm range were collected using the Jasco Spec V750 spectrophotometer and 10 mm pathlength cuvette. To observe changes in the spectral properties of bound heme at increasing temperatures, UV−Vis spectra were collected at each 10 °C interval between 25 °C and 95 °C. Temperature was increased at the rate of 5 °C min^{-1}, and spectra were acquired after the temperature had stabilized to within 0.5 °C of target temperature for 5 seconds. Measurements were performed with 20 μM solutions of purified holoprotein in TBS buffer (25 mM Tris-HCl, 300 mM NaCl, pH 8.2). The collected spectra are presented in Figure S3 and Figure S5.

Circular dichroism spectroscopy. To determine secondary structure and thermostability of the designs, far-ultraviolet circular dichroism (CD) measurements were carried out on a JASCO J-1500 instrument using a 1 mm pathlength cuvette. Samples of purified protein were prepared at 1.0 mg/mL in 20 mM sodium phosphate, 50 mM potassium fluoride, pH 8.0 (TEV protease) or at 0.4 mg/mL in 25 mM Tris, 20 mM NaCl, pH 8.2 (myoglobin). The temperature of the sample was ramped from 25 °C to 95 °C with full spectrum scans from 190 nm to 260 nm performed after each 10 °C interval. The signal at 216 nm was plotted over the temperature gradient and fitted to a Boltzmann sigmoidal curve with GraphPad Prism 9. T_m values were calculated from the inflection point.

Mass spectrometry analysis. MS data for myoglobin variants were acquired on an Agilent 1200series LC G6230B TOF LC-MS with an AdvanceBio RP-Desalting column (A: H2O with 0.1% Formic Acid, B: Acetonitrile with 0.1% Formic Acid). The final protein concentrations were adjusted to 1-2 mg/mL in 25 mM Tris-HCl, 300 mM NaCl, pH 8.2. Subsequent data deconvolution was performed in Bioconfirm using a total entropy algorithm. All data are presented in Table S2.

Molecular dynamics simulations. Structures generated with AlphaFold2[8] were used as starting geometries. For the protein-substrate complexes, substrate peptide was superimposed onto AlphaFold2 structures using the crystallographic structure of catalytically active TEV protease (PDB: 1LVM) as a template. Simulations were carried out with AMBER 20[9] implemented with the ff14SB force field for the protein and substrate peptide, and the general Amber force field (GAFF2)[10] for the substrate peptide C-terminal fluorescent probe (7-amino-4-(trifluoromethyl)coumarin). Parameters were generated with the *antechamber* module of AMBER, combining ff14SB and GAFF2 force fields and with partial charges set to fit the electrostatic potential generated with HF/6-31G(d) using the RESP method.[11] The charges were calculated according to the Merz-Singh-Kollman scheme using Gaussian 16.[12] Binding-site histidine residue (H46) was modeled in its Nδ1-H tautomeric state (corresponding to residue name HID in Amber). Initial structures were neutralized with either Na^+ or Cl^- ions and set at the center of a cubic TIP3P[13] water box with a buffering distance between solute and box of 10 Å.

A two-stage geometry optimization approach was performed. The first stage minimizes only the positions of solvent molecules and ions, and the second stage is an unrestrained minimization of all the atoms in the simulation cell. The system was then heated by incrementing the temperature from 0 to 300 K under a constant pressure of 1 atm and periodic boundary conditions (PBC). Harmonic restraints of 10 kcal/mol were applied to the solute, and the Andersen temperature coupling scheme[14,15] was used to control and equalize the temperature. The time step was kept at 1 fs during the heating stages, allowing potential inhomogeneities to self-adjust. Water molecules were treated with the SHAKE algorithm[16] such that the angle between the hydrogen atoms was kept fixed through the simulations. Long-range electrostatic effects were

modeled using the particle mesh Ewald method.[17] An 8 Å cut-off was applied to Lennard-Jones interactions. The system was equilibrated for 2 ns with a 2 fs time step at a constant volume and temperature of 300 K. Ten independent production trajectories were then run for additional 1000 ns under the same simulation conditions, leading to accumulated simulation times of 10 μs for each system. Root mean square (rms) fluctuations and interatomic distance analyses were carried out with the *cpptraj* module of AMBER.

Sequence alignments. Alignments of designs and their parent sequences were generated using Clustal Omega[18] with default settings.

COMPUTATIONAL DETAILS

Myoglobin backbone idealization with inpainting. The backbone idealization of Rosetta-relaxed crystal structure of human myoglobin (PDB: 3RGK) was performed using RoseTTAFold joint inpainting.[19] Two separate design trajectories were performed. In first, the following regions were considered for idealization: 9 N-terminal residues, 10 C-terminal residues, positions 73-88 connecting the E and F helices. In the second strategy, in addition to the above, positions 47-59 in the CD-loop region were considered for remodeling (Figure 2A). Furthermore, positions in the fixed parts of the protein that are in contact with the remodeled regions and are not part of the heme binding site were allowed to be redesigned using the "inpaint_seq" option.

The following settings were included in the input JSON files to perform the design:

Strategy 1:

```
[{"pdb": "../3RGK_fr.pdb",
"task": "hal",
"dump_all": true,
"inf_method": "multi_shot",
"n_cycle": 15,
"num_designs": 20,
"tmpl_conf": "0.9",
"contigs": ["6-10,A10-72,14-19,A89-139,8-12"],
"inpaint_seq": ["A130","A134","A137"],
"out": "3RGK_inpaint1"}]
```

Strategy 2:

```
[{"pdb": "../3RGK_fr.pdb",
"task": "hal",
"dump_all": true,
"inf_method": "multi_shot",
"n_cycle": 15,
"num_designs": 10,
"tmpl_conf": "0.9",
"contigs": ["6-10,A10-46,10-16,A60-72,14-19,A89-139,8-12"],
"inpaint_seq": ["A26","A30","A34","A62","A130","A134","A137"],
"out": "3RGK_inpaint2"}]
```

ProteinMPNN design of myoglobin. The following command was used to perform ProteinMPNN[20] sequence redesign of the native myoglobin as well as the structures obtained from inpainting backbone idealization.

```
python $MPNN_PATH/protein_mpnn_run.py --jsonl_path ../parsed_pdbs_bb.jsonl --
fixed_positions_jsonl ../masked_pos.jsonl --batch_size 1 --out_folder ./ --num_seq_per_target
20 --sampling_temp "0.1 0.2 0.3" --omit_AAs='MC' --checkpoint_path
$MPNN_PATH/vanilla_model_weights/v_48_020.pt
```

Where `parsed_pdbs_bb.jsonl` contains the parsed PDB file information, created with the script `$MPNN_PATH/helper_scripts/parse_multiple_chains.py`

`masked_pos.jsonl` file contains the positions that were kept fixed during sequence design:

{"3RGK": {"A": [39, 42, 43, 45, 64, 67, 68, 71, 72, 89, 92, 93, 97, 99, 104, 107, 138]}}

For each of the outputs from the inpainting backbone idealization, the fixed position numbers were readjusted to correspond to the positions in the parent structure.

ProteinMPNN design of TEV protease. The following command was used to perform sequence design with ProteinMPNN on TEV protease.

```
python $MPNN_PATH/protein_mpnn_run.py \
         --jsonl_path ../parsed_pdbs_bb.jsonl \
         --chain_id_jsonl ../assigned_chains.jsonl \
         --fixed_positions_jsonl ../masked_pos.jsonl \
         --out_folder $MPNN_OUTDIR \
         --num_seq_per_target 16 \
         --sampling_temp "0.1 0.2 0.3" \
         --batch_size 8 \
         --omit_AAs='XC'
```

Where `../assigned_chains.jsonl` contains the parsed PDB chain information: `{"TEVd": [["A"]]}`

Sets of designs were distinguished by selection of fixed residues.

Designs with only the amino acid identities of the active site fixed during sequence design had the following residues fixed:

[31, 32, 44, 46, 81, 134, 135, 139, 146, 147, 148, 149, 150,151, 167, 168, 169, 170, 171, 172, 173, 174, 175, 176, 177, 178,204, 208, 209, 211, 213, 214, 215, 216, 217, 218, 219, 220]

Designs with the amino acid identities of active site residues and the 30% most conserved residues fixed during sequence design had the following residues fixed:

[3, 7, 9, 10, 11, 12, 14, 19, 25, 34, 36, 38, 42, 44, 46, 47, 48, 51, 52, 53, 55, 61, 62, 64, 68, 81, 88, 89, 90, 92, 94, 100, 101, 103, 110, 113, 116, 117, 126, 127, 129, 139, 140, 142, 143, 144, 146, 149, 151, 152, 154, 156, 160, 161, 163, 165, 167, 169, 177, 186, 190, 198, 202, 211, 212, 221]

Designs with the amino acid identities of active site residues and the 50% most conserved residues fixed during sequence design had the following residues fixed:

[2, 3, 7, 8, 9, 10, 11, 12, 13, 14, 21, 23, 25, 26, 27, 31, 32, 34, 35, 36, 37, 38, 41, 42, 43, 44, 46, 47, 48, 51, 52, 53, 55, 59, 61, 62, 64, 68, 70, 72, 76, 81, 85, 88, 89, 90, 91, 92, 93, 94, 95, 98, 100, 101, 103, 107, 109, 112, 113, 115, 116, 117, 119, 123, 125, 126, 127, 129, 133, 134, 135, 139, 140, 141, 142, 143, 144, 146, 147, 148, 149, 150, 151, 152, 153, 154, 156, 157, 160, 161, 163, 165, 167, 168, 169, 170, 171, 172, 173, 174, 175, 176, 177, 178, 179, 182, 183, 186, 190, 198, 200, 202, 204, 205, 208, 209, 211, 212, 213, 214, 215, 216, 217, 218, 219, 220, 221]

Designs with the amino acid identities of active site residues and the 70% most conserved residues fixed during sequence design had the following residues fixed:

```
[1, 2, 3, 4, 7, 8, 9, 10, 11, 12, 13, 14, 15, 18, 21, 22, 23, 25, 26, 27, 31, 32, 33, 34, 35,
36, 37, 38, 40, 41, 42, 43, 44, 46, 47, 48, 49, 50, 51, 52, 53, 55, 57, 59, 61, 62, 63, 64,
66, 68, 69, 70, 71, 72, 73, 76, 79, 80, 81, 83, 84, 85, 86, 87, 88, 89, 90, 91, 92, 93, 94,
95, 96, 97, 98, 100, 101, 103, 107, 108, 109, 111, 112, 113, 115, 116, 117, 118, 119, 120,
122, 123, 124, 125, 126, 127, 129, 131, 133, 134, 135, 137, 139, 140, 141, 142, 143, 144,
145, 146, 147, 148, 149, 150, 151, 152, 153, 154, 155, 156, 157, 158, 160, 161, 163, 164,
165, 166, 167, 168, 169, 170, 171, 172, 173, 174, 175, 176, 177, 178, 179, 182, 183, 186,
187, 189, 190, 194, 196, 198, 200, 202, 203, 204, 205, 206, 207, 208, 209, 211, 212, 213,
214, 215, 216, 217, 218, 219, 220, 221]
```

SUPPLEMENTARY FIGURES

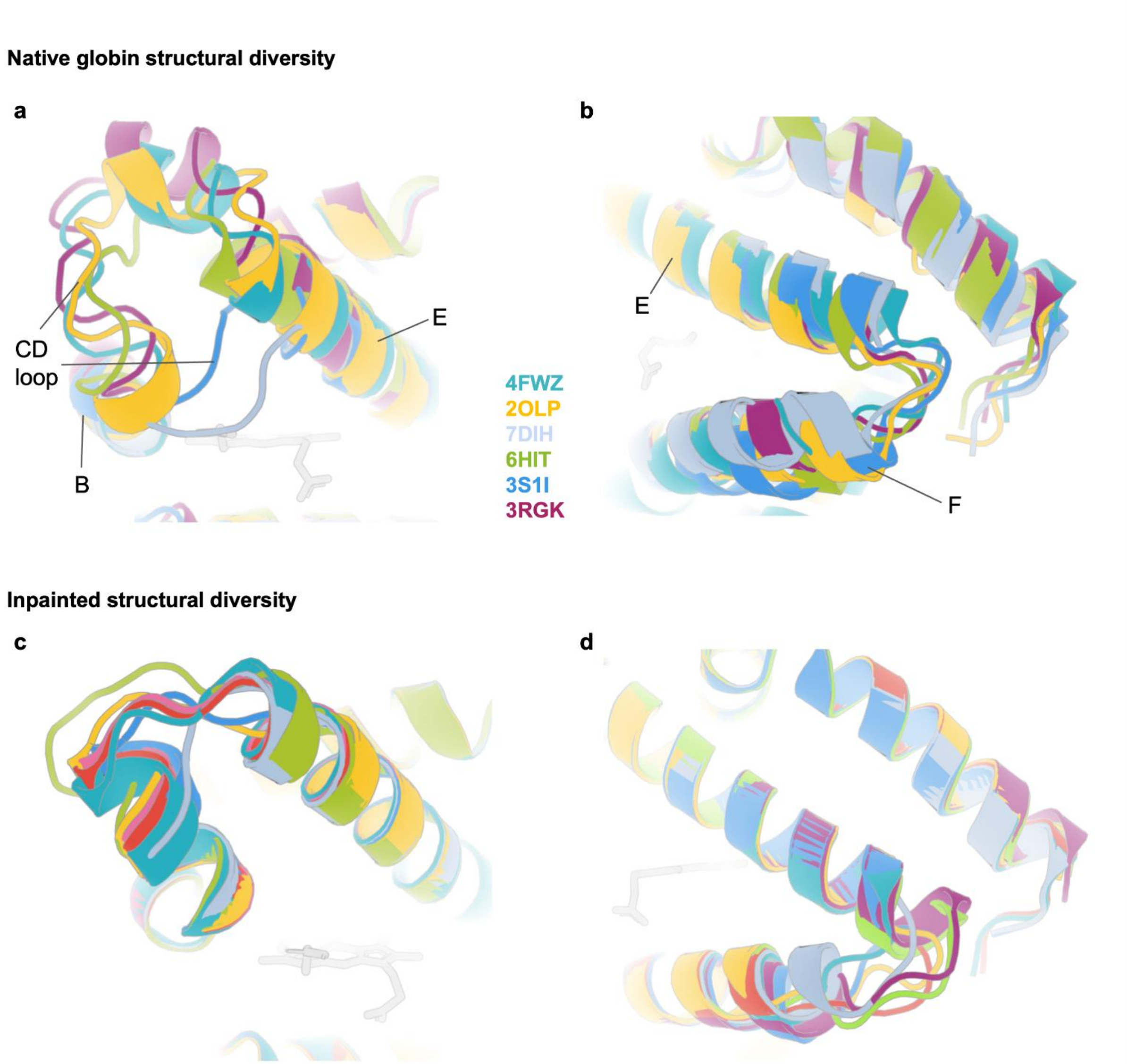

Figure S1. Inpainting samples different backbone structures compared to native globins. (A) Diversity of the CD-loop region in selected native globins. (B) Diversity in the loop connecting helices E and F in selected native globins. (C) Diversity of inpainted motifs replacing the CD-loop region. (D) Diversity of inpainted loops connecting helices E and F.

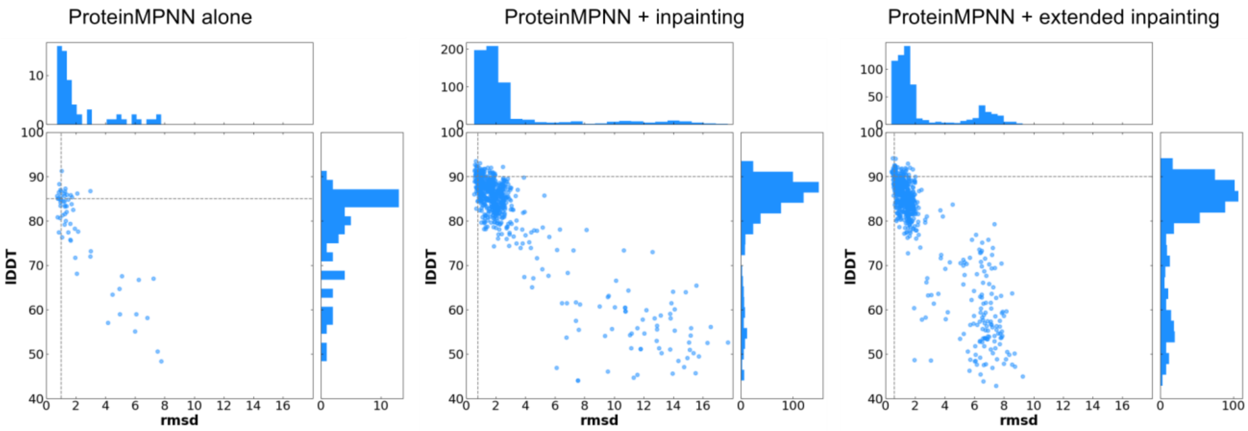

Figure S2. Extensive backbone remodeling with RoseTTAFold joint inpainting improves structure prediction metrics. Designs made with only sequence redesign had the lowest-scoring structure prediction metrics (lDDT and RMSD to design model) amongst all designs, while designs subjected to the most aggressive backbone remodeling strategy scored the highest in these metrics. Dashed lines indicate lDDT and RMSD cutoffs used for design selection, with the top left sector containing successful designs.

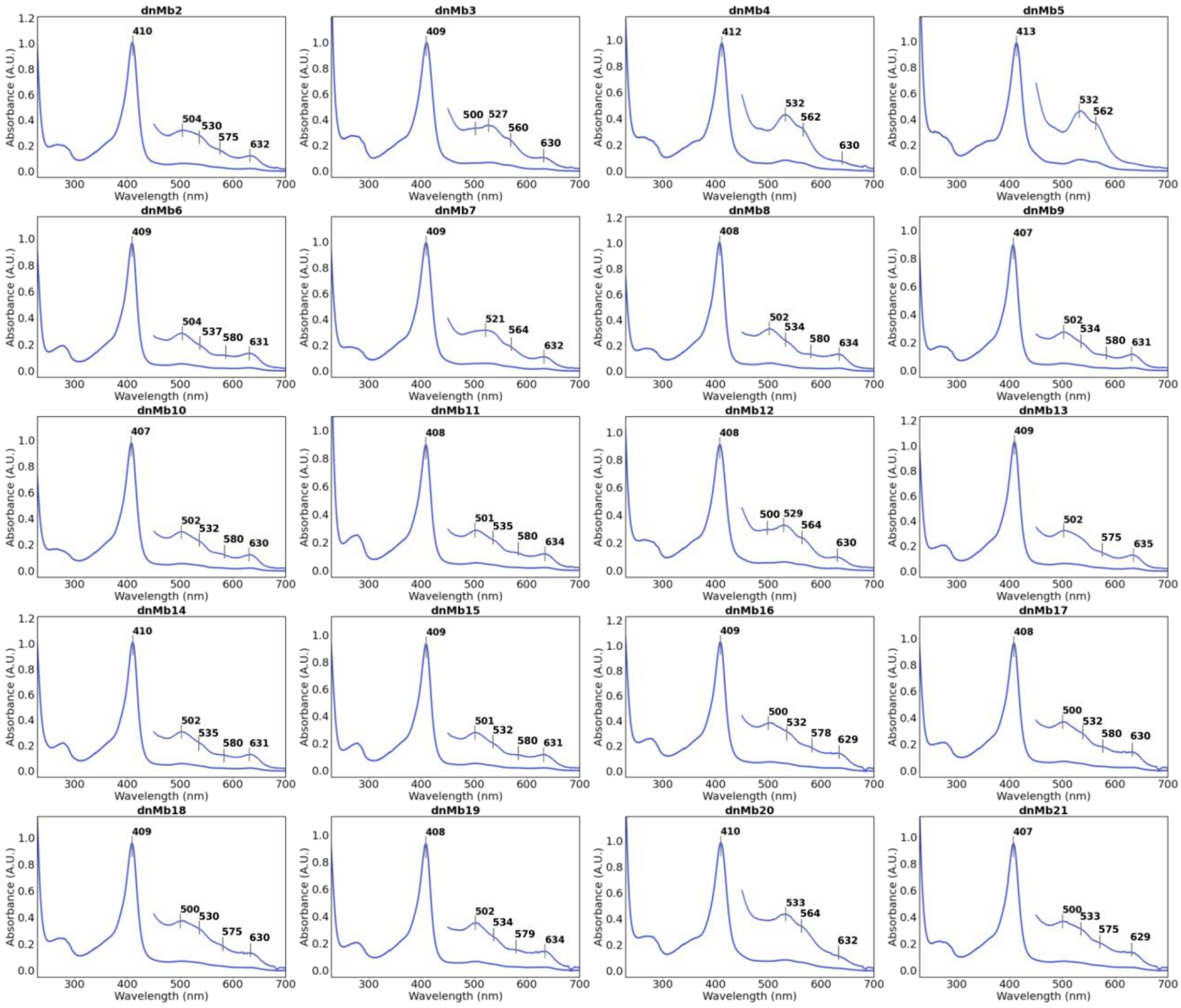

Figure S3. UV/Vis spectra of myoglobin variants. Spectroscopic data of most designs is in close agreement with that of native myoglobin (Soret maximum at 409 nm; Q band features at 500, 537, 582 and 630 nm), suggesting pentacoordinate heme-binding. A few designs (dnMb3, dnMb4, dnMb5, dnMb12, and dnMb20) show some degree of hexacoordinate heme-binding (potentially through incorporation of imidazole from the

purification buffer), indicated by the major Q band features at ~530 and ~560 nm. Spectra were recorded in a buffer containing 25 mM Tris-HCl and 300 mM NaCl at pH 8.2.

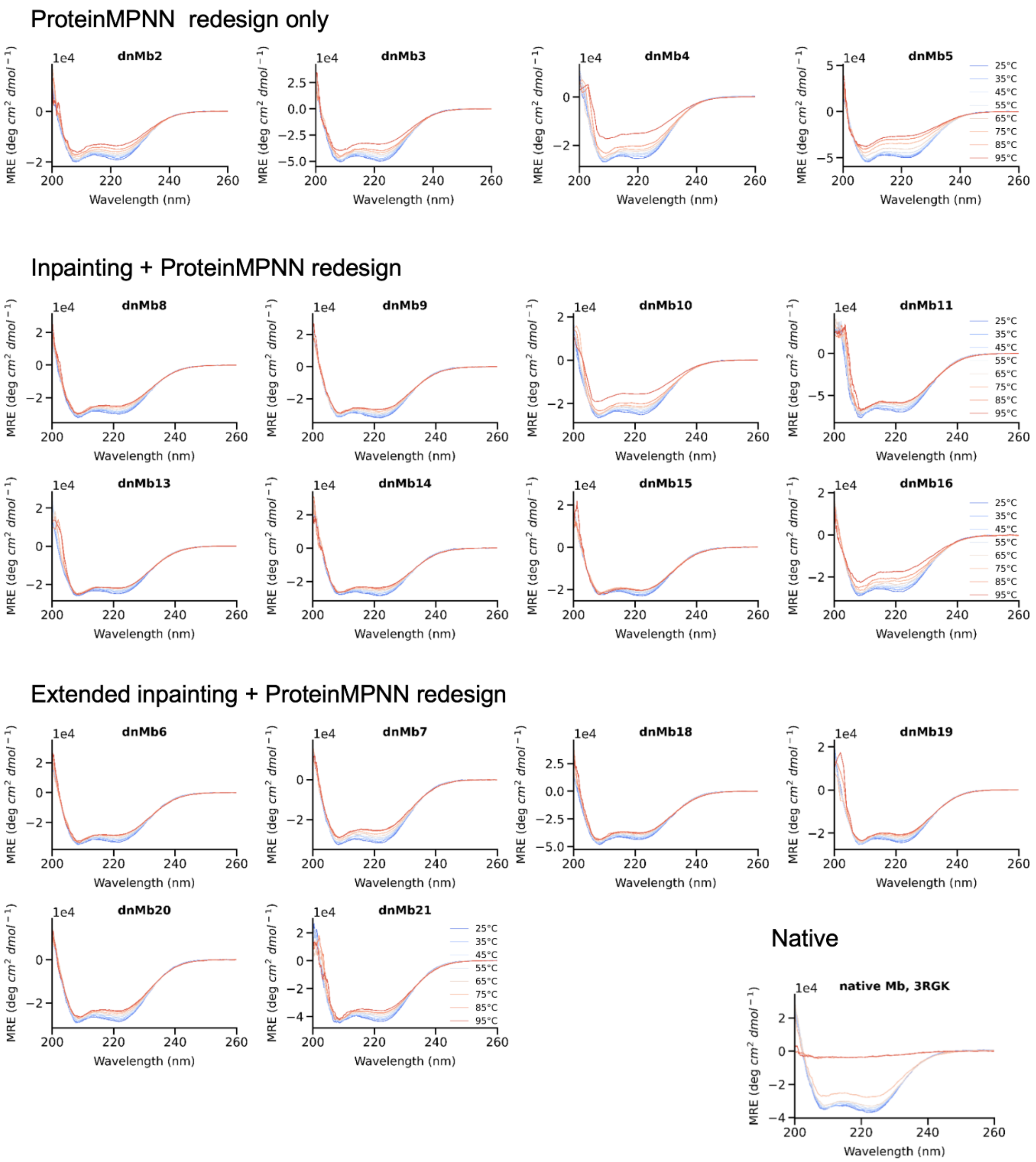

Figure S4. Many myoglobin designs show increased thermostability over parent. CD spectroscopy signal of myoglobin designs and parent sequence nMb over a temperature gradient from 25 °C to 95 °C indicates elevated resistance to unfolding in designs. CD signal reported in molar residue ellipticity (MRE).

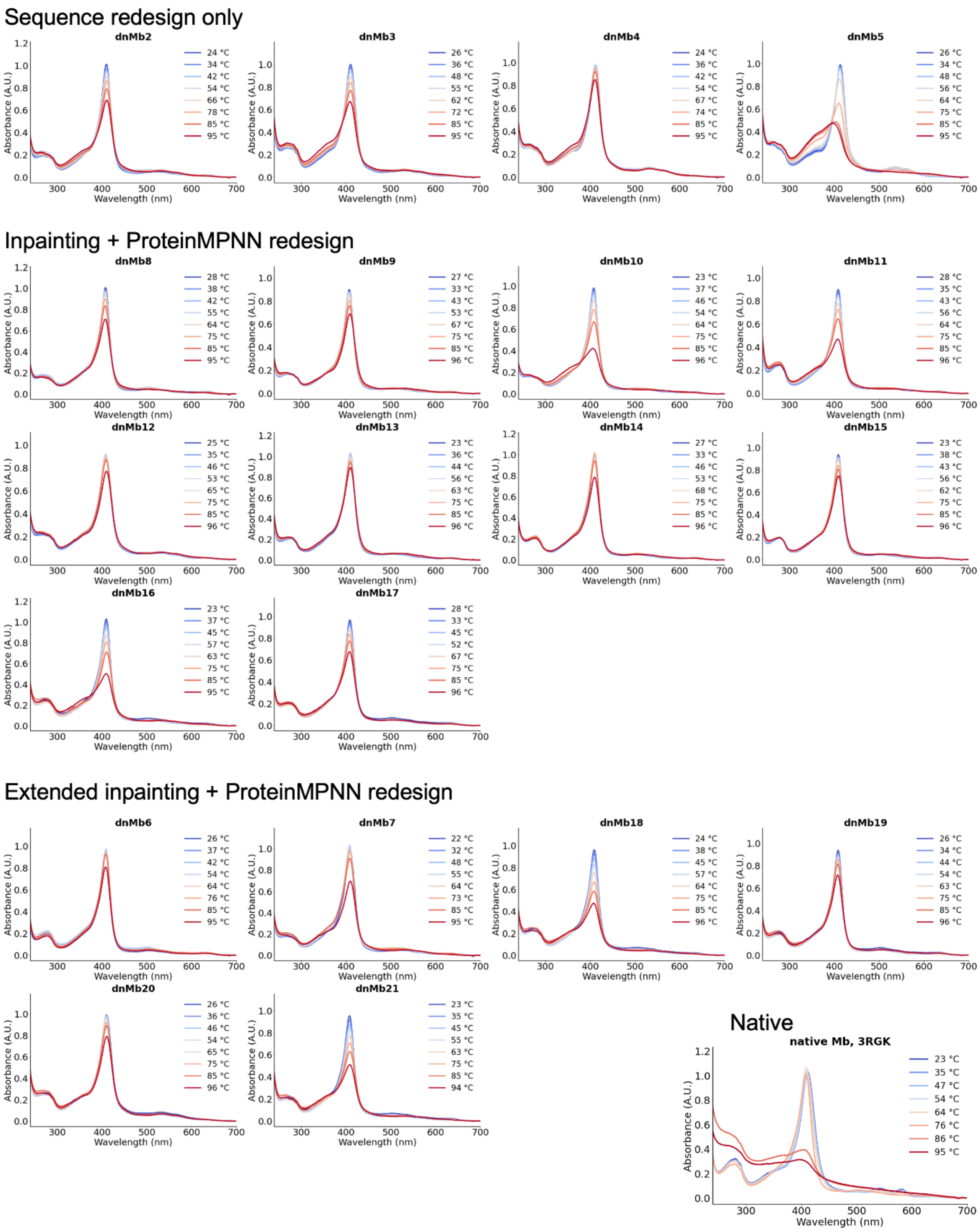

Figure S5. Myoglobin designs retain heme binding at higher temperatures than parent. Heme binding as measured by UV/Vis absorbance over a temperature gradient from 25 °C to 95 °C indicates retention of function at higher temperatures in designs. Higher melting temperatures of designs indicate more temperature-stable binding sites.

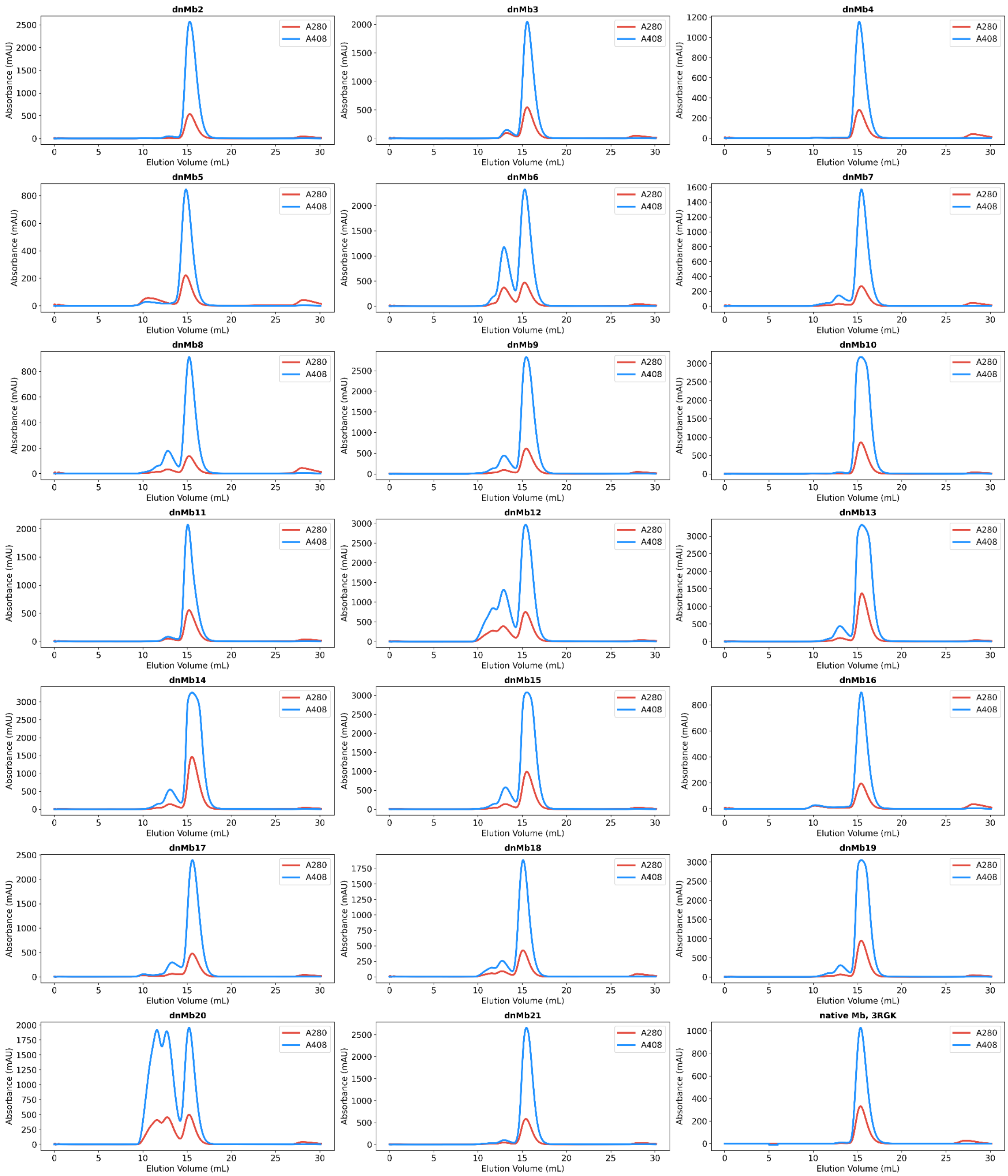

Figure S6. Size-exclusion chromatograms of heme-loaded myoglobin variants. Data were collected using a Superdex Increase 75 10/300 GL column (GE Healthcare) in a buffer containing 25 mM Tris-HCl and 300 mM NaCl at pH 8.2. Void volume of the column is 8.5 mL. Blue chromatograms were obtained by following the absorbance at 408 nm, indicating elution of heme-containing species. Red chromatograms were obtained from absorbance at 280 nm.

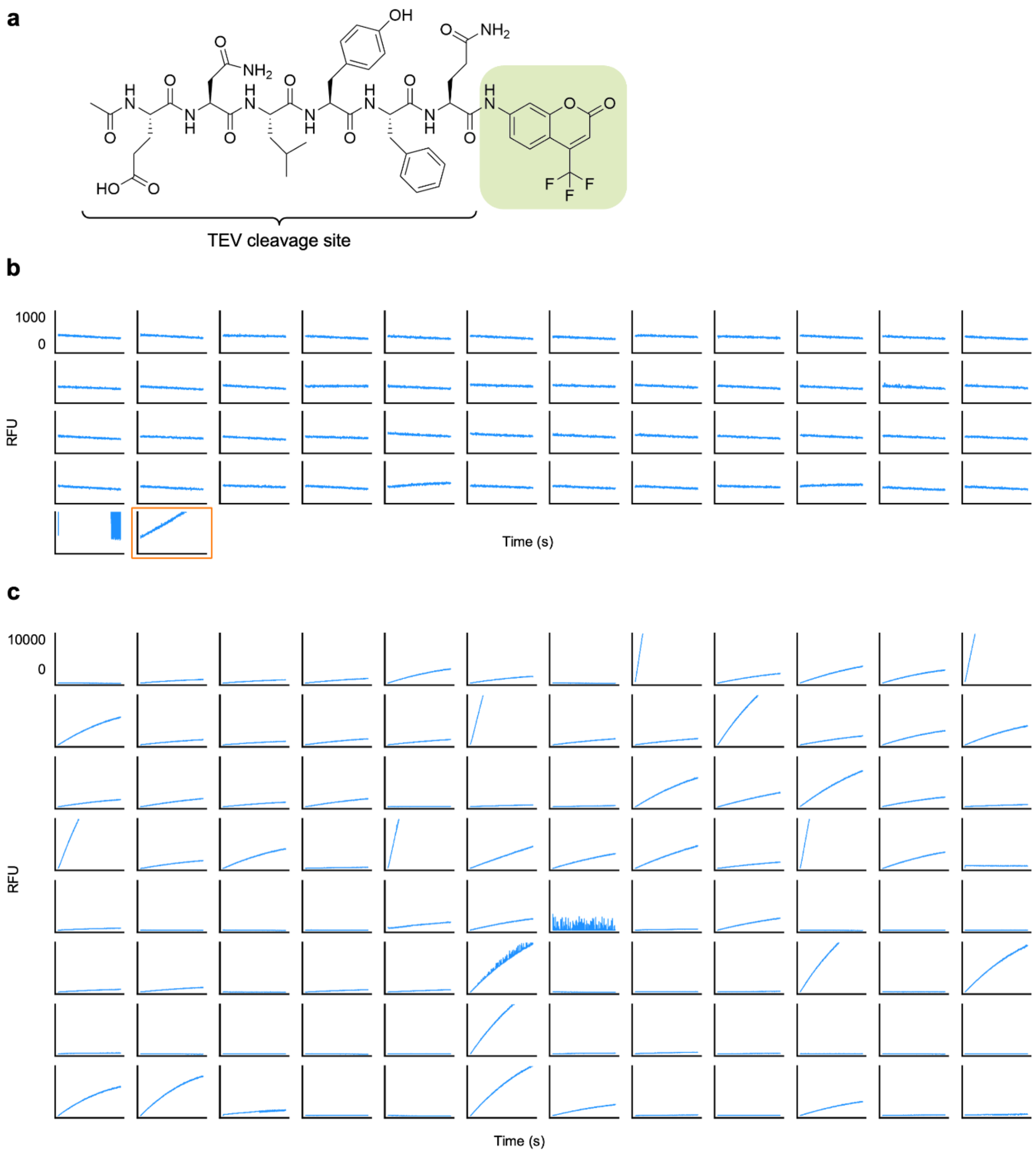

Figure S7. Initial screen of proteolytic activity on fluorescent reporter substrate. Pure protein was normalized to 1 μM and assayed against 10 μM substrate, AFC, in an initial screen for catalytic turnover. (A) Structure of the peptide-coumarin substrate, AFC, used to assay proteolytic activity. (B) Raw fluorescence data (in raw fluorescence units, RFU) for designs generated with only active site residues fixed or with active site residues and 30% most conserved residues fixed during design. TEVd plot outlined in orange. (C) Raw fluorescence data for designs generated with active site residues fixed and 50% most conserved residues fixed or with active site residues fixed and 70% most conserved residues fixed.

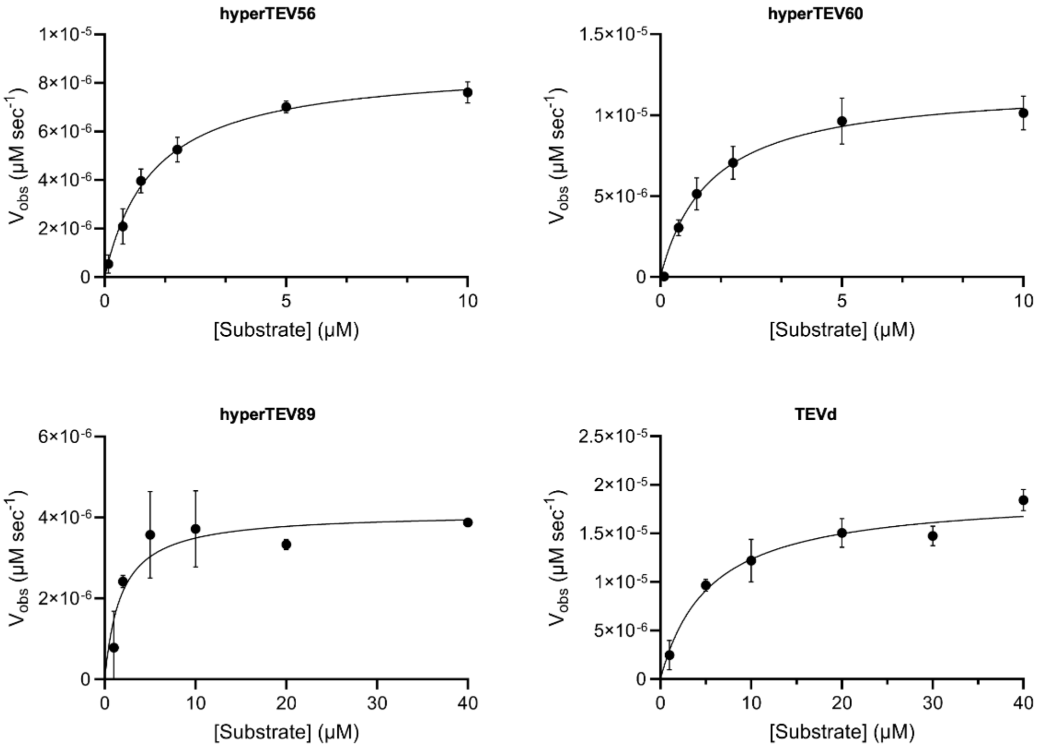

Figure S8. Michaelis Menten kinetics of TEV redesigns and parent. Michaelis Menten plots for three TEV designs and TEVd. Error bars represent standard deviation from three technical replicates. hyperTEV designs were assayed at 50 nM while TEVd was assayed at 500 nM.

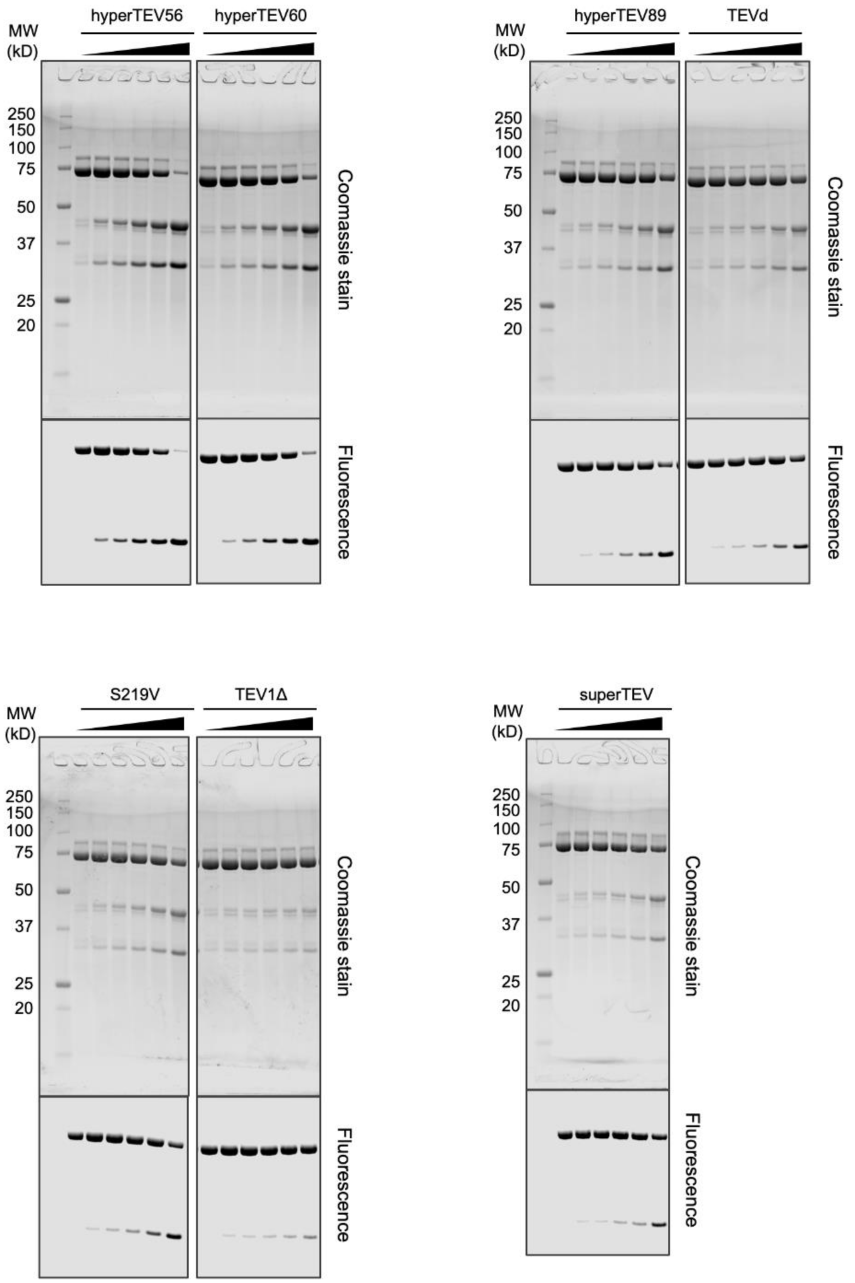

Figure S9. SDS-PAGE gels of protein substrate cleavage by TEV designs. Protein standard molecular weight ladder is shown on the left, with molecular weight markers indicated in kD. For each gel, the coomassie-stain is shown above and the EGFP fluorescence image is shown below.

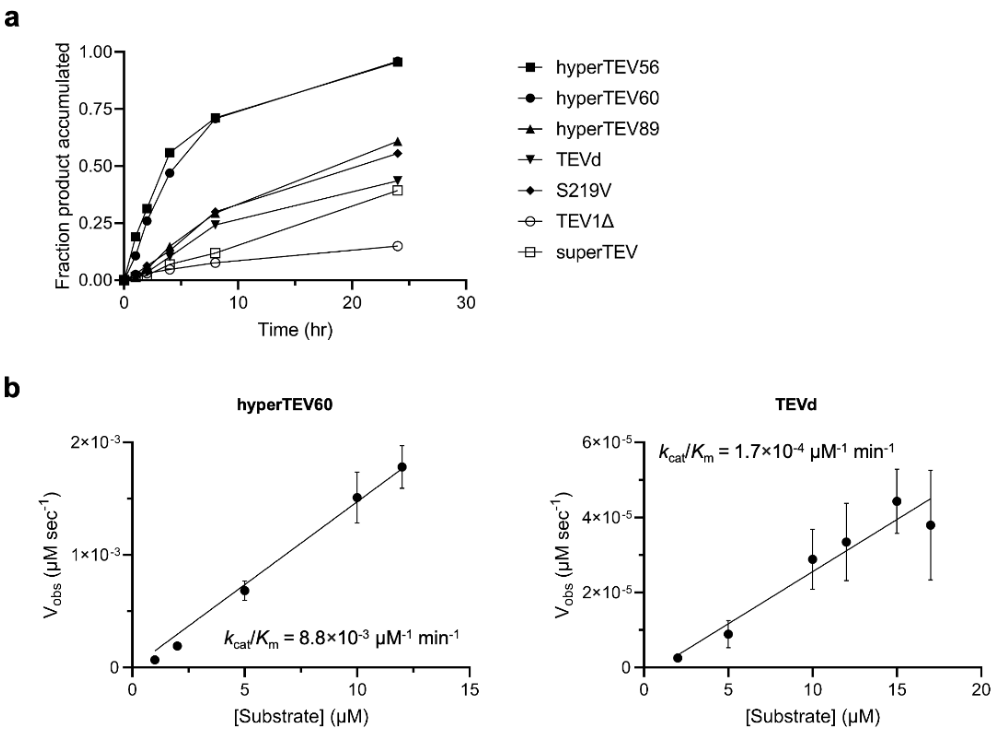

Figure S10. Activity of TEV redesigns in a gel-based activity assay. (A) Plot of accumulated product normalized to fluorescence intensity of uncleaved substrate over time. Fluorescence intensity was quantified with ImageJ software. Designs hyperTEV56 and hyperTEV60 show increased turnover rate compared to reported TEV variants. (B) Straight-line fit for initial turnover rates in gel assay for hyperTEV60 and TEVd. Curves were fitted from monitoring of substrate depletion for hyperTEV60 and production accumulation for TEVd. Error bars represent standard deviation from three technical replicates.

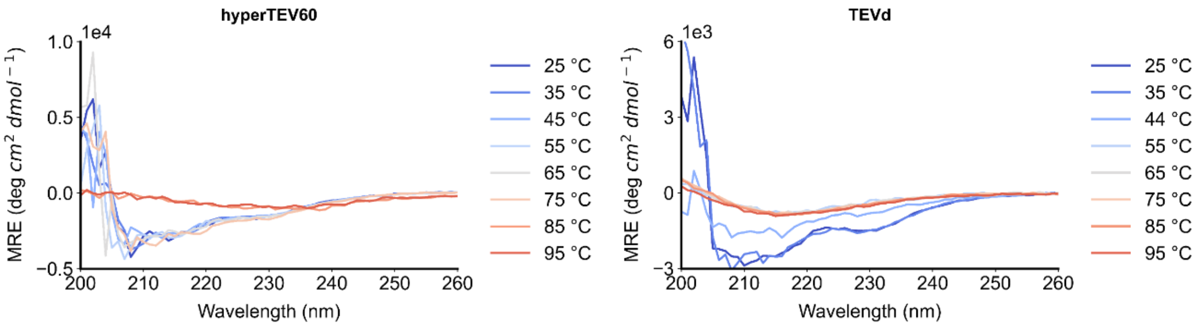

Figure S11. TEV design shows increased thermostability over parent. CD spectroscopy signal of hyperTEV60 and TEVd over a temperature gradient from 25 °C to 95 °C indicates elevated resistance to unfolding in ProteinMPNN design hyperTEV60. CD signal reported in molar residue ellipticity (MRE).

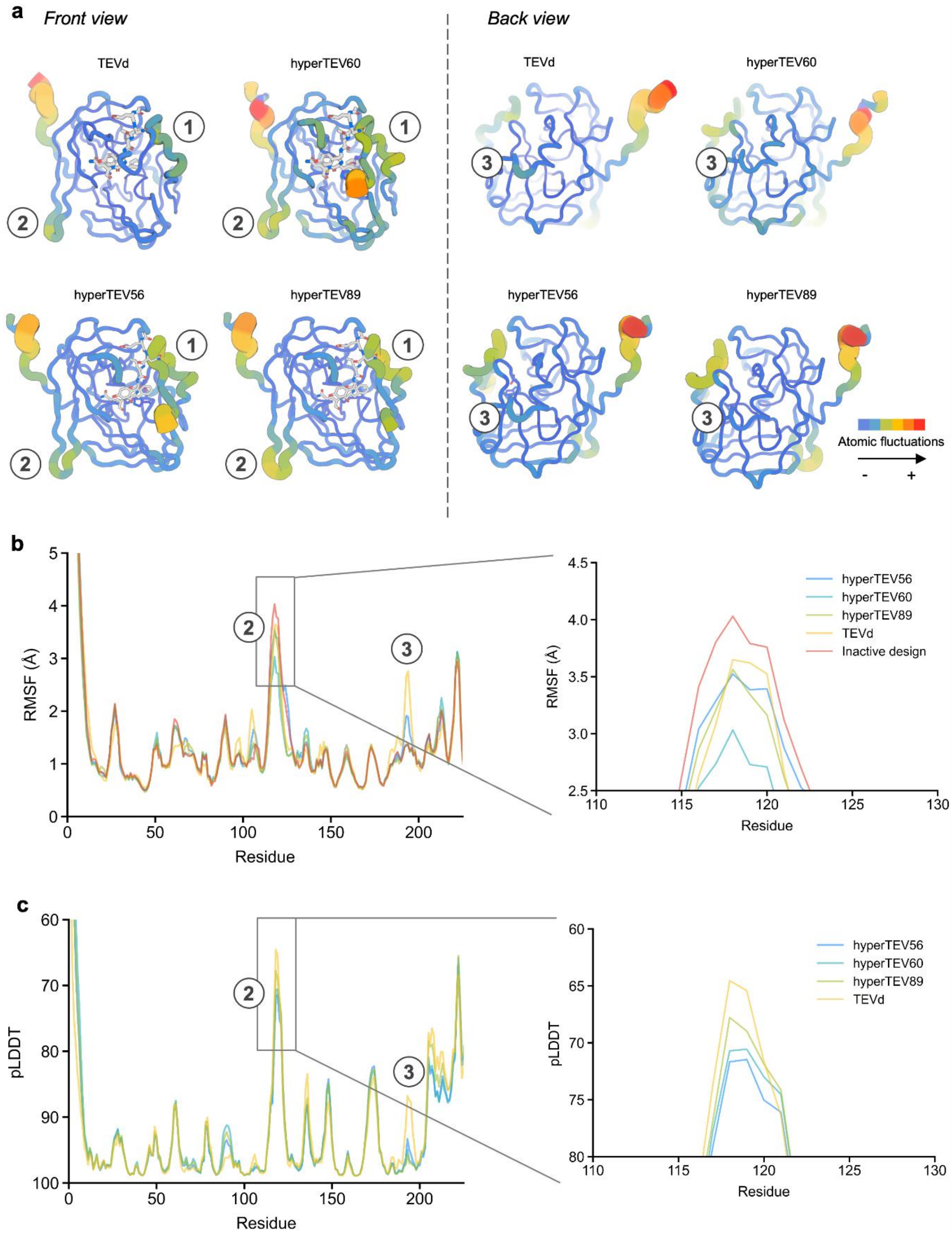

Figure S12. Designs show trends in rigidification and activity in molecular dynamics simulations. (A) Molecular dynamics (MD) simulations revealed trends of rigidification of several loops (marked with numbered circles) in the redesigned structures as compared to the parent. Directly adjacent to the peptide binding site, region 1 (residues 206-215) shows diminishing mobility in hyperTEV60 and other redesigns as compared to TEVd. An internal loop designated as region 3 (residues 192-194) shows significant loss of atomic fluctuation relative to TEVd. (B) Cα root mean square fluctuation (RMSF) of designs in region 2 (residues 115-124) denoted in (A) shows a positive correlation between activity and rigidification, with TEVd and a design inactive on the peptide substrate showing most flexibility in this region. (C) Per-residue pLDDT values from AlphaFold2 ensemble prediction exhibit similar trends of increased rigidification in more active designs.

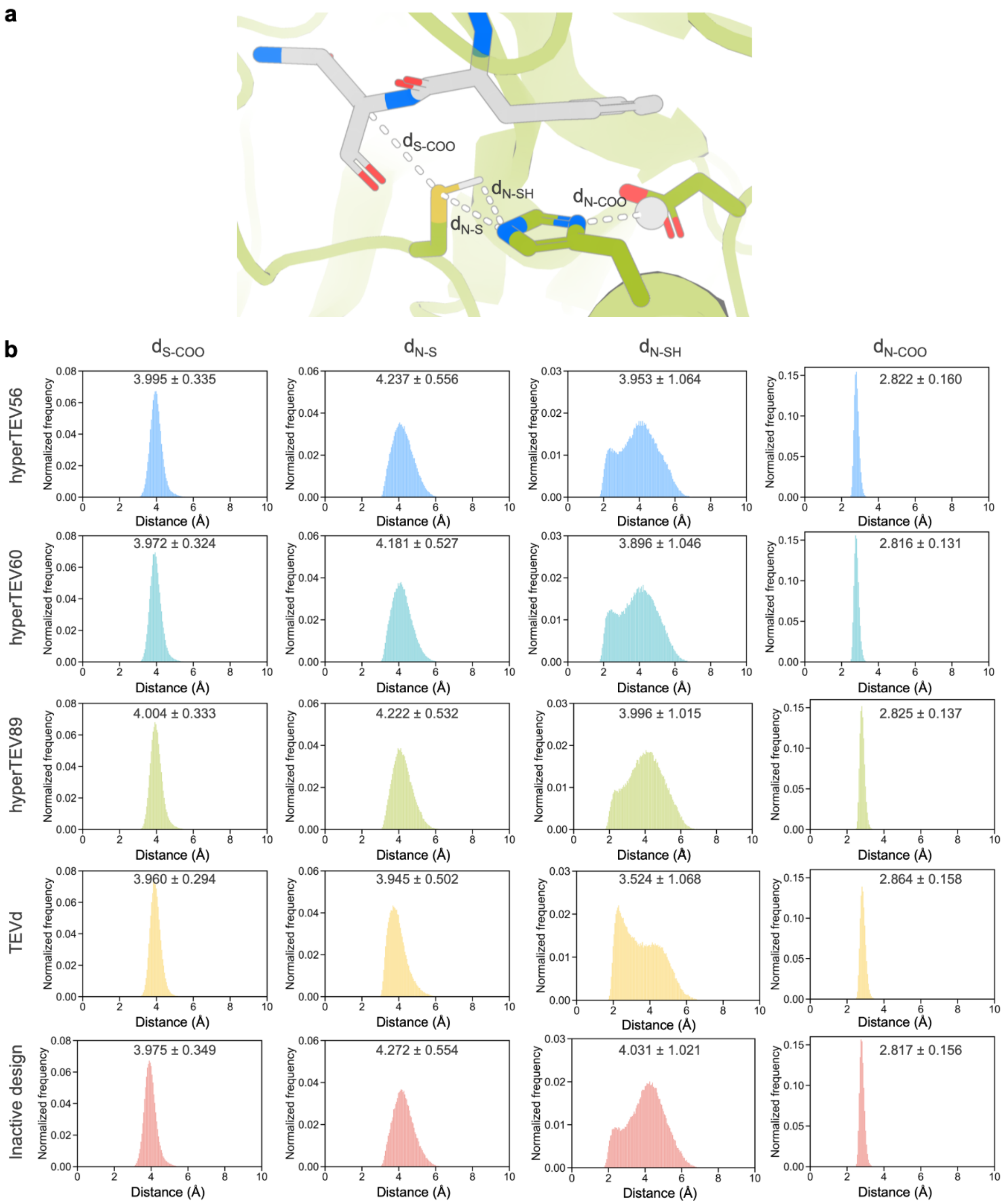

Figure S13. Population of catalytically competent dyad conformers correlates with activity in MD simulations. (A) Key distances in the TEV catalytic triad as shown on TEVd. Peptide substrate is shown in gray. (B) Distances of key interactions in the catalytic triad were measured across MD simulations. Average distances for each interaction in each TEV variant are inset. Catalytically competent conformers of the Cys-His dyad ($d_{N\text{-}SH}$) are less populated in designs as compared to TEVd. Among designs, the highest activity variant hyperTEV60 has the highest percentage of competent dyad conformers.

Table S1. Myoglobin sequence similarity analysis against UniRef100

Variant	Design method	Sequence similarity with parent (3RGK)	Highest sequence similarity	Most similar UniRef100 ID
dnMb2	ProteinMPNN only	47%	51%	P02182
dnMb3	ProteinMPNN only	49%	52%	UPI00148EC9BB
dnMb4	ProteinMPNN only	51%	55%	P02182
dnMb5	ProteinMPNN only	46%	51%	Q0KIY3
dnMb6	Inpaint CD+EH; ProteinMPNN	40%	44%	A0A8C5V5K1
dnMb7	Inpaint CD+EH; ProteinMPNN	42%	48%	UPI001C20C4EB
dnMb8	Inpaint EH; ProteinMPNN	45%	47%	A0A8C9A9W3
dnMb9	Inpaint EH; ProteinMPNN	46%	53%	UPI001C20C4EB
dnMb10	Inpaint EH; ProteinMPNN	46%	51%	UPI00148EC9BB
dnMb11	Inpaint EH; ProteinMPNN	49%	55%	P02185
dnMb12	Inpaint EH; ProteinMPNN	51%	54%	A0A4W2F1N8
dnMb13	Inpaint EH; ProteinMPNN	44%	49%	UPI001CA46E1B
dnMb14	Inpaint EH; ProteinMPNN	46%	51%	A0A8C9A9W3
dnMb15	Inpaint EH; ProteinMPNN	46%	50%	UPI000011026E
dnMb16	Inpaint EH; ProteinMPNN	39%	45%	P02169
dnMb17	Inpaint EH; ProteinMPNN	42%	45%	F6PMG4
dnMb18	Inpaint CD+EH; ProteinMPNN	42%	43%	R9RZ90
dnMb19	Inpaint CD+EH; ProteinMPNN	39%	41%	UPI0003C8C8C2
dnMb20	Inpaint CD+EH; ProteinMPNN	45%	48%	P02182
dnMb21	Inpaint CD+EH; ProteinMPNN	39%	44%	P02182

Table S2. Mass spectrometry data for myoglobin variants.

Variant	Expected mass	Observed mass
dnMb2 (Met missing)	19186	19054
dnMb3 (Met missing)	18981	18850
dnMb4 (Met missing)	19054	18923
dnMb5 (Met missing)	18441	18310
dnMb6 (Met missing)	19604	19473
dnMb7 (Met missing)	18728	18597
dnMb8 (Met missing)	19604	19472
dnMb9 (Met missing)	19579	19448
dnMb10 (Met missing)	18690	18559
dnMb11 (Met missing)	19464	19333
dnMb12 (Met missing)	19536	19405
dnMb13 (Met missing)	19178	19047
dnMb14 (Met missing)	19675	19544
dnMb15 (Met partially missing)	19598	19467,19598
dnMb16 (Met partially missing)	19441	19310,19441
dnMb17 (Met missing)	20247	20115
dnMb18 (Met partially missing)	19427	19296,19427
dnMb19 (Met missing)	19452	19321
dnMb20 (Met partially missing)	18814	18683,18814
dnMb21 (Met missing)	19133	19002
nMb 3RGK (Met missing)	18751	18620

Table S3. The extinction coefficients of the Soret band and R_z values (A_{Soret} / A_{280}) of myoglobin variants.

Variant	Extinction coefficient (mM^{-1} cm^{-1})	R_z
dnMb2	128 ± 3	5.7
dnMb3	166 ± 15	4.0
dnMb4	127 ± 1	4.6
dnMb5	154 ± 8	4.9
dnMb6	181 ± 14	5.4
dnMb7	159 ± 6	5.8
dnMb8	186 ± 2	6.8
dnMb9	182 ± 5	5.5
dnMb10	157 ± 2	7.4
dnMb11	177 ± 8	4.1
dnMb12	123 ± 3	4.9
dnMb13	154 ± 2	5.2
dnMb14	174 ± 1	4.8
dnMb15	156 ± 1	4.9
dnMb16	171 ± 4	4.5
dnMb17	170 ± 1	5.4
dnMb18	175 ± 15	3.1
dnMb19	171 ± 3	5.1
dnMb20	150 ± 4	3.7
dnMb21	153 ± 1	4.6

CRYSTALLOGRAPHIC DATA

The protein sample for crystallography was prepared following the general procedure for myoglobin production. The holoprotein was purified using Ni-affinity and size exclusion chromatography. The C-terminal hexahistidine tag was left intact. The holo dnMb19 was crystallized at 17 mg mL^{-1} in a buffer containing 25 mM Tris-HCl, 300 mM NaCl, pH 8.2.

The crystallization experiment for the designed protein was conducted using the sitting drop vapor diffusion method. Crystallization trials were set up in 200 nL drops using the 96-well plate format at 20°C. Crystallization plates were set up using a Mosquito LCP from SPT Labtech, then imaged using UVEX microscopes from JAN Scientific. Diffraction quality crystals formed in 0.1 M Bis-Tris pH 6.5, 28% w/v Polyethylene glycol monomethyl ether 2,000 (Index crystallization screen, Hampton Research, well D11).

Diffraction data were collected at ALS-ENABLE beamline 8.2.2. X-ray intensities and data reduction were evaluated and integrated using XDS[21] and merged/scaled using Pointless/Aimless in the CCP4 program suite[22]. Structure determination and refinement starting phases were obtained by molecular replacement using Phaser[23] using the designed model structure. Following molecular replacement, the models were improved using phenix.autobuild[24]. Structures were refined in Phenix[24]. Model building was performed using COOT[25]. The final model was evaluated using MolProbity[26]. Data collection and refinement statistics are recorded in Table S4. Data deposition, atomic coordinates, and structure factors reported for the protein in this paper have been deposited in the Protein Data Bank (PDB), http://www.rcsb.org/ with accession code 8U5A.

Table S4. Crystallographic statistics for dnMb19.

	**dnMb19**
PDB accession number	8U5A
Wavelength (Å)	1.0
Resolution range	42.64 - 2.0 (2.05 - 2.0)
Space group	P 1 2_1 1
Unit cell dimensions<br>a, b, c, (Å)<br>α, β, γ (°)	 31.589 41.669 128.439<br>90 95.13 90
Unique reflections	22200 (1513)
Multiplicity	4.3 (4.1)
Completeness (%)	97.30 (95.52)
Mean I/sigma(I)	10.95 (1.58)
Wilson B-factor	36
R-merge	0.07531 (0.9919)
R-pim	0.04041 (0.5512)
CC1/2	0.997 (0.773)
Reflections used in refinement	22200 (1513)
R-work	0.2301 (0.3312)
R-free	0.2581 (0.3741)
Number of non-hydrogen atoms	2612
macromolecules	2440
ligands	87
solvent	85
Protein residues	298
RMS(bonds)	0.002
RMS(angles)	0.39
Ramachandran favored (%)	99.32
Ramachandran allowed (%)	0.68
Ramachandran outliers (%)	0

Average B-factor	44
macromolecules	44.03
ligands	40.92
solvent	46.5

SEQUENCE INFORMATION

Alignment of TEV hit sequences

```
TEVd            GESLFKGPRDYNPISSTICHLTNESDGHTTSLYGIGFGPFIITNKHLFRRNNGTLLVQSL   60
hyperTEV89      AESAAPGPRDYNPISSTIVRLTNTSDGHSISLFGIGFGPLIITNAHLFRRNNGTLTITSL   60
hyperTEV56      MESAAPGPRDYNPISDTIVKLTNTSDGYSISLYGIGFGPLIITNAHLFRRNNGTLTVTSK   60
hyperTEV60      AESAAPGPRDYNPISDTIVLLTNTSDGYSISLYGIGFGPLIITNAHLFRRNNGTLTITSK   60
                 **   *********.**  *** ***:: **:******:**** ********** : *

TEVd            HGVFKVKNTTTLQQHLIDGRDMIIIRMPKDFPPFPQKLKFREPQREERICLVTTNFQTKS   120
hyperTEV89      HGTFTISNTTTLKLHLIEGRDLVIIKMPKDFPPFPTTLEFREPVVGEDIVLVTRNFQDKD   120
hyperTEV56      HGTFTIENTTTLQLHLIEGRDLVIIKMPKDFPPFPTDLVFREPVEGEKITLVTRNFQTKE   120
hyperTEV60      HGTFTISNTTTLKLHLIEGRDLVLIEMPKDFPPFPTNLVFREPVVGEEIVLVTRNFQTKT   120
                **.*.:.*****: ***:***:::*.*********  * ****   * * *** *** *

TEVd            MSSMVSDTSCTFPSSDGIFWKHWIQTKDGQCGSPLVSTRDGFIVGIHSASNFTNTNNYFT   180
hyperTEV89      PTSEVSDTSTTEPSSDGVFWKHWIPTKDGQCGSPMVSVSDGSIVGIHSASNFTNTNNYFT   180
hyperTEV56      PTSEVSDVSSTYPSSDGVFWKHWIPTKDGQCGSPMVSVEDGSIVGIHSASNFTNTNNYFT   180
hyperTEV60      PTSEVSDVSTTYPSSDGVFWKHWIPTKDGQCGSPMVSVTDGSIVGIHSASNFTNTNNYFT   180
                 :* ***.* * *****:****** *********:**. ** ******************

TEVd            SVPKNFMELLTNQEAQQWVSGWRLNADSVLWGGHKVFMDKP  221
hyperTEV89      AVPPNFMDLLTDPSLQKWISGWSLNADSVDWGGHKVFMDKP  221
hyperTEV56      AVPPDFMDLLTNDSLQKWISGWSLNSDSVEWGGHKVFMDKP  221
hyperTEV60      AVPPDFMRLLTDPSLQKWVSGWSLNSDSVEWGGHKVFMDKP  221
               :** :** ***: . *:*:*** **:*** ***********
```

Alignment of myoglobin sequences

```
3RGK        -GLSDGEWQLVLNVWGKVEADIPGHGQEVLIRLFKGHPETLEKFDRFKHLKSEDEMKA-S      58
dnMb02      -GLTEEEQKLVWEIFERFEEDLEGFGLDVLIRAFTEHPETLKKFPRFADLKSEAELRA-S      58
dnMb03      -GLTAEEQKLVRDIWAEVEKDREGFGLEVLLLTFTEHPETLKKFPRFAHLKSAEELRA-S      58
dnMb04      -GLTAEEQALVRAIWAKVREDLEGFGLAVLLKTFTEHPETLKKFPRFKDLKSEEEILA-S      58
dnMb05      -GLSDEEQALVLSIFEKVKEDLAGFGLDVLLLAFTKNPATLEKFPRFADLKSEAELLA-S      58
dnMb20      --LSEEEWKIVLEIFALVREDLAGVGAAVLERTFATHPETLKKFPRFLAAAEAGVLD--R      56
dnMb16      ---AEEEKEKVLSIFKLVEKDKKTIGSEVLIITFTKNPETKKKFPRFKDLKTVEELKA-S      56
dnMb19      ---SEEKAALVLALFDRVEADREEIGAAVLRRTFEEHPETLKKFPRFLELYKKGSPEL-D      56
dnMb18      ---DEEKKKLVLEAFELVEKDIEGIGAEVLKLTFEKHPETLEKFPRLKELHAAGSPEL-E      56
dnMb15      ---EEEKQIVLELFAKVEEDLEGIGLEVLILTFTKHPETRKKFPRFAHLTTEAQLQA-S      56
dnMb17      IKLSEEEKKLVLEIFKLFEENLEEFGKEVLITTFTKHPETKKKFPRFAHLKTEEEFLA-S      59
dnMb13      ----DERNKLVLSAFALVREDLEEIGAEVLILTFTENPETLKKFPRFAHLKTEEELKK-S      55
dnMb14      ---EKEKNELVLKAFELIEKDLEGFGSEVLILTFTKHPETLKKFPRFKHLKTEEEFKA-S      56
dnMb21      -SLTPEELAIVKALFARVREDLEGVGAEVLRLTFEKHPETLKKFPRFLELKKAGSPEL-E      58
dnMb07      -KLTPEEKAIVLRIFALVREDRAGIGAAILRRTFEAHPETLEKFPRLRALRAAGREAELE      59
dnMb06      -KLSEEEKEIVLKIFELVEKDVEEIGLRVLELTFEKHPETLEKFPRLRELLAAGRLEELE      59
dnMb10      ---DAEKQALVASIFAKFEADLEGFGKAVLIKTFTKHPETRKKFPRFKHLKSVEELEK-S      56
dnMb11      -KLSEEEKEIVLKIFALVEKDLEGFGKEVLIKTFLKYPETLKKFPRFKHLKTEEELKA-S      58
dnMb12      LNLSPEDKAKVLEIFALVEEDLEGFGREVLILTFTKHPETLKKFPRFAHLKTEEELRA-S      59
dnMb08      IKISEEEFEIVLEIFELVKKDLAGIGKEVLILTFTKHPETLKKFPRFAHLKTVEELEA-S      59
dnMb09      SKLTEEEWKTVFKIFALVEKDLEGFGLAVLIRTFTRYPETLKKFPRFAHLKTVEELRA-S      59
                      *   :  .. :    *  :*   *   * * :** *:

3RGK        EDLKKHGATVLTALGGI---LKKKGHHEAEIKPLAQSHATKHKIPVKYLEFISEAIIQVL      115
dnMb02      PRLREHGVTVLKALIKI---FKKGEDFAEEVKPLAESHSKVHKIPVSDLEVIAAAILATA      115
dnMb03      PEAKAHGVTVLDALSKI---LKKGSNFEEEIKPLAESHYKEHKIPIEDLKVIADAIIAVL      115
dnMb04      EKAKKHGVTVLTALFAI---FDKGENFEEEIKPLAESHYKEHKIPISDLKVIADAIVAVL      115
dnMb05      EKAKEHGITVLTALFAI---FEKGDDFDAEVEPLATSHTREHKIPTSDLEVIAAAILETA      115
dnMb20      ALLAAHGETVLTALIEIAES----KLDPELIKKLAESHVKEHKIPIEYLRAIADSLIAVL      112
dnMb16      EKVKDHGVTVLDALIEWARLHVEGKDYDSLVKKLAESHKKEHKIPIEDLKSIADALIEVL      116
dnMb19      ALLKEHGKTVLDALIEIARLRYSGEDYRSLIKELAKSHKEEHKIPIEDLRHIAEALLAVL      116
dnMb18      ELLKEHGATVLKALIEIARLKISGGDYLSLVKELAKSHKEEHKIPIEDLKKIAEALLEVL      116
dnMb15      PELKQHGVTVLKALITIAKLYYEGKDYESLIKELAKSHKEEHKIPIEYLEYISESILEVL      116
dnMb17      PELAKHGVTVLNALIEIAKLYLEGKDYRSLIKKLAKSHKLEHKIPIEDLKYIADAIIEVA      119
dnMb13      PLLKEHGVTVLNALIEIAELKYSGGDYESLVKELAKSHKEKHKIPIEDLKAIAEAILKVL      115
dnMb14      EELKEHGVTVLKALIEIAKLKVSGEDYDSLIKELAKSHKTKHKIPIEYLKYIADAILEVA      116
dnMb21      AELRAHGVTVLTALIELADNY---EGNNETLEKLAESHTKVHKIPVSDLKNIAAAIIEVL      115
dnMb07      ALLREHGVTVLDALIEI---V--ENDDEELLKKLAESHKTTHKIPIEHLEHIAAALLEVL      114
dnMb06      AYLREHGVTVLKALIEA---I--KNEDEELLEKLAKSHKEEHKIPIEYLKYIADSIIEVL      114
dnMb10      EELKEHGVTVLTALREIS--L--GENQDKKIKDLATSHKEKHKIPIEDLEVIAAAILEVA      112
dnMb11      EELKEHGVTVLKALIEI---F--KNEDEEKLKELAKSHKEEHKIPIEDLEKIAEAIIEVL      113
dnMb12      EELKEHGVTVLKALRAI---L--EKGDEELLKKLAESHTKEHKIPVSDLEVIAESIIEVA      114
dnMb08      PLLAEHGVTVLKALIKIVEEL--KKGDTSLIKELAKSHKTEHKIDIKDLKYIAESIIEVL      117
dnMb09      PLLREHGVTVLKALTKIAEEL--KKGKTGTLKKLAESHSKVHKIPISDLERIAEAIIEVL      117
                 ** *** **                 :. ** **   ***  . *. *: ::: .

3RGK        QSKHPGDFGADAQGAMNKALELFRKDMASNYKEL-      149
dnMb02      KERFPEFFNEKAQAALTKALQQFIDAIAAEYKKL-      149
dnMb03      KKRFPTAFNSAAQAAVTKALQQFIDALEKEFKKL-      149
dnMb04      KEAFPEAFDAKAQAAFTKALEQFIKAFEEEYKKL-      149
dnMb05      KERFPTEFDEEAQAALEKALAQFIAAYAAQAAKL-      149
```

```
dnMb20      KERYPERFGEKAQEAVKKFLDLFIEKFEEEAEKEK    147
dnMb16      KKFYPEEFGEEAQAAVQKLLNYFIEKLKQYYE---    148
dnMb19      AERFPDEFGPEARAALTDFLDWFIAEIEEEYKK--    149
dnMb18      KEKYPEEFGEETQEALKEFLDWFIEELEKEFKE--    149
dnMb15      KKRFPEFFGEKAQAAVRKFLDFFISKLKEYYE---    148
dnMb17      KKFFPEKFGEKAQEALKKFLNYFIEELEKEYEKL-    153
dnMb13      KKRYPEEFGEKTQAALKEFLDEFIELTEKYYK---    147
dnMb14      KKRFPKEFDEKTYAALKEFLDYFIEKIEKYYK---    148
dnMb21      KERFPEEFGEEAQAAFTKFLDKFIKDIAELQKKFE    150
dnMb07      AEKYPEEFGPEARAAVTKALELFIKKLAEFYE---    146
dnMb06      EEKFPKEFNEKAREALKKALEYFIEELEKYYK---    146
dnMb10      KERFPEEFDEAAQAALQEFLDDFISKLKEYFE---    144
dnMb11      KEKYPEEFDEEAEEAVKKFLKLFIEKLKEYRE---    145
dnMb12      KKRFPEEFGEEAQAALKKFLEEFIEKWKEYQEEFK    149
dnMb08      KKRFPEEFDEKAKEAVEKVLNLFIEKIEEFYKK--    150
dnMb09      EERFPEEFDEKAKEAVKKFLDLFIEKHAEFVKK--    150
             . .*  *.  :  *. . *  *
```

Myoglobin sequences

dnMb2

```
ATGTCAGGAGGCCTGACCGAAGAAGAACAGAAACTGGTGTGGGAAATTTTTGAACGCTTTGAAGAGGATCTGGAAGGCTTTGGCCTGGATGTG
CTGATTCGCGCGTTTACCGAACATCCAGAAACCCTGAAAAAATTTCCGCGCTTTGCGGATCTGAAAAGCGAAGCGGAATTACGTGCGAGCCCG
CGCCTGCGCGAACATGGCGTGACCGTGCTGAAAGCGCTGATTAAAATCTTTAAAAAAGGCGAAGATTTTGCCGAAGAAGTGAAACCGCTGGCG
GAAAGCCATAGCAAAGTGCATAAAATTCCGGTGAGCGATCTCGAAGTGATTGCGGCGGCGATTCTGGCGACCGCGAAAGAACGCTTTCCGGAA
TTTTTTAATGAAAAAGCGCAGGCGGCGCTGACCAAAGCACTGCAGCAGTTTATTGATGCGATCGCCGCGGAATATAAAAAACTGGGCGGCGGT
TCCGGCAGCCATCATTGGGGCAGCACCCACCACCACCACCAC
```

```
MSGGLTEEEQKLVWEIFERFEEDLEGFGLDVLIRAFTEHPETLKKFPRFADLKSEAELRASPRLREHGVTVLKALIKIFKKGEDFAEEVKPLA
ESHSKVHKIPVSDLEVIAAAILATAKERFPEFFNEKAQAALTKALQQFIDAIAAEYKKLGGGSGSHHWGSTHHHHHH
```

dnMb3

```
ATGTCAGGAGGCCTGACCGCGGAAGAACAGAAACTGGTGCGCGATATTTGGGCGGAAGTGGAAAAAGATCGCGAAGGCTTTGGCCTGGAAGTG
CTGTTGCTGACCTTTACCGAACATCCAGAAACCTTAAAAAAATTTCCACGTTTTGCGCATCTGAAAAGCGCCGAGGAACTGCGCGCGAGCCCG
GAAGCGAAAGCGCATGGCGTGACCGTGCTGGATGCGCTGAGCAAAATTTTGAAGAAAGGCAGCAATTTCGAAGAAGAAATTAAACCGCTGGCG
GAAAGCCATTATAAAGAACATAAAATTCCGATTGAAGATTTGAAAGTGATTGCGGATGCGATTATTGCGGTGCTGAAAAAACGCTTTCCGACC
GCGTTTAATAGCGCGGCGCAGGCGGCGGTGACCAAAGCGCTGCAGCAGTTTATTGATGCACTGGAAAAGGAATTTAAGAAACTGGGCGGCGGT
TCCGGCAGCCATCATTGGGGCAGCACCCACCACCACCACCAC
```

```
MSGGLTAEEQKLVRDIWAEVEKDREGFGLEVLLLTFTEHPETLKKFPRFAHLKSAEELRASPEAKAHGVTVLDALSKILKKGSNFEEEIKPLA
ESHYKEHKIPIEDLKVIADAIIAVLKKRFPTAFNSAAQAAVTKALQQFIDALEKEFKKLGGGSGSHHWGSTHHHHHH
```

dnMb4

```
ATGTCAGGAGGTTTAACCGCGGAAGAACAAGCGCTGGTGCGCGCGATTTGGGCGAAAGTGCGCGAAGATCTGGAAGGCTTTGGCCTGGCGGTG
CTGCTGAAAACCTTTACCGAACATCCGGAAACCCTGAAAAAATTTCCGCGCTTTAAAGACTTGAAAAGCGAAGAAGAAATTCTGGCGAGCGAA
AAGGCGAAAAAACATGGCGTGACCGTGCTGACTGCGCTGTTTGCGATTTTTGATAAAGGTGAGAATTTTGAAGAGGAAATTAAACCGCTGGCG
GAAAGCCATTATAAAGAACATAAAATTCCGATTAGCGATCTGAAAGTGATTGCGGATGCGATTGTGGCCGTGTTGAAAGAAGCGTTTCCGGAA
GCATTTGATGCGAAAGCGCAGGCGGCGTTTACCAAAGCACTGGAACAGTTTATTAAAGCGTTCGAGGAAGAATATAAAAAACTGGGCGGCGGT
TCCGGCAGCCATCATTGGGGCAGCACCCACCACCACCACCAC
```

```
MSGGLTAEEQALVRAIWAKVREDLEGFGLAVLLKTFTEHPETLKKFPRFKDLKSEEEILASEKAKKHGVTVLTALFAIFDKGENFEEEIKPLA
ESHYKEHKIPISDLKVIADAIVAVLKEAFPEAFDAKAQAAFTKALEQFIKAFEEEYKKLGGGSGSHHWGSTHHHHHH
```

dnMb5

```
ATGTCAGGAGGCCTGAGCGATGAAGAACAGGCGCTGGTGCTGAGCATTTTTGAAAAAGTGAAAGAAGATCTGGCGGGCTTTGGCCTGGATGTG
CTGTTGCTGGCGTTTACCAAAAATCCGGCGACCCTGGAAAAATTTCCGCGCTTTGCGGATCTGAAAAGCGAAGCGGAACTGTTGGCGAGCGAA
AAGGCCAAAGAACATGGCATTACCGTGCTGACCGCGCTGTTTGCGATTTTCGAGAAAGGCGATGATTTTGATGCGGAAGTTGAACCGCTGGCG
```

ACCAGCCATACCCGCGAACATAAAATTCCGACGAGCGATCTGGAAGTGATTGCGGCGGCGATTCTGGAAACCGCGAAGGAACGCTTTCCAACC
GAATTTGATGAAGAAGCGCAGGCGGCCTTAGAAAAAGCGTTGGCGCAGTTTATTGCAGCGTATGCGGCGCAAGCCGCGAAACTGGGCGGCGGT
TCCGGCAGCCATCATTGGGGCAGCACCCACCACCACCACCACCAC

MSGGLSDEEQALVLSIFEKVKEDLAGFGLDVLLLAFTKNPATLEKFPRFADLKSEAELLASEKAKEHGITVLTALFAIFEKGDDFDAEVEPLA
TSHTREHKIPTSDLEVIAAAILETAKERFPTEFDEEAQAALEKALAQFIAAYAAQAAKLGGGSGSHHWGSTHHHHHH

dnMb6

ATGTCAGGAAAACTGAGCGAAGAAGAAAAAGAAATTGTGCTGAAAATTTTTGAACTGGTGGAAAAGGATGTGGAAGAAATTGGCCTGCGCGTG
CTGGAACTGACCTTTGAAAAACATCCAGAAACCCTGGAGAAATTTCCACGCTTACGCGAATTATTAGCGGCGGGCCGCCTGGAGGAACTGGAA
GCGTATCTGCGCGAACATGGCGTGACCGTGTTAAAAGCGCTGATTGAAGCGATTAAAAATGAAGATGAAGAACTGTTGGAAAAACTGGCGAAA
AGCCATAAAGAGGAACATAAAATTCCGATTGAATATCTGAAATATATTGCGGATAGCATTATTGAAGTGTTAGAAGAGAAGTTTCCGAAAGAA
TTTAATGAAAAGGCGCGCGAAGCGTTGAAGAAAGCACTGGAATATTTTATTGAGGAGCTGGAGAAATATTATAAAGGCGGCGGTTCCGGCAGC
CATCATTGGGGCAGCACCCACCACCACCACCACCAC

MSGKLSEEEKEIVLKIFELVEKDVEEIGLRVLELTFEKHPETLEKFPRLRELLAAGRLEELEAYLREHGVTVLKALIEAIKNEDEELLEKLAK
SHKEEHKIPIEYLKYIADSIIEVLEEKFPKEFNEKAREALKKALEYFIEELEKYYKGGGSGSHHWGSTHHHHHH

dnMb7

ATGTCAGGAAAATTAACCCCAGAAGAAAAAGCGATTGTTTTACGTATTTTTGCGTTAGTTCGTGAAGATCGTGCGGGTATTGGTGCGGCGATT
TTGCGTCGTACCTTTGAAGCGCATCCAGAAACCTTAGAAAAATTTCCACGTTTACGTGCGTTACGCGCCGCGGGCCGCGAAGCGGAACTGGAA
GCGCTGTTGCGTGAACATGGCGTGACCGTGCTGGATGCGCTGATTGAAATTGTGGAAAATGATGATGAAGAACTGCTGAAAAAACTGGCGGAA
AGCCATAAAACCACCCACAAAATTCCAATTGAACATTTAGAACATATTGCGGCGGCGCTGCTGGAAGTGCTGGCCGAGAAATATCCGGAAGAA
TTTGGTCCGGAGGCGCGCGCAGCGGTGACCAAAGCCTTGGAACTGTTTATTAAAAAGCTCGCGGAATTTTATGAAGGCGGCGGTTCCGGCAGC
CATCATTGGGGCAGCACCCACCACCACCACCACCAC

MSGKLTPEEKAIVLRIFALVREDRAGIGAAILRRTFEAHPETLEKFPRLRALRAAGREAELEALLREHGVTVLDALIEIVENDDEELLKKLAE
SHKTTHKIPIEHLEHIAAALLEVLAEKYPEEFGPEARAAVTKALELFIKKLAEFYEGGGSGSHHWGSTHHHHHH

dnMb8

ATGTCAGGAATTAAAATTAGCGAAGAAGAATTTGAAATTGTGCTGGAAATTTTTGAACTGGTGAAAAAAGATCTGGCGGGCATTGGCAAAGAA
GTGCTGATTCTGACCTTTACCAAACATCCAGAAACCCTGAAGAAATTTCCACGTTTTGCGCATCTGAAAACCGTGGAAGAACTGGAAGCGAGC
CCGCTGCTGGCGGAACATGGCGTGACCGTGCTGAAAGCGCTGATTAAGATCGTGGAGGAACTGAAGAAAGGCGATACCAGCCTGATCAAAGAA
CTGGCGAAAAGCCATAAAACCGAACATAAGATTGATATTAAGGATTTGAAATATATTGCGGAAAGCATTATTGAAGTTTTAAAAAAACGCTTT
CCGGAAGAGTTCGATGAAAAAGCGAAAGAAGCGGTGGAAAAAGTGTTGAATCTGTTTATCGAGAAAATCGAAGAATTTTATAAAAAGGGCGGC
GGTTCCGGCAGCCATCATTGGGGCAGCACCCACCACCACCACCACCAC

MSGIKISEEEFEIVLEIFELVKKDLAGIGKEVLILTFTKHPETLKKFPRFAHLKTVEELEASPLLAEHGVTVLKALIKIVEELKKGDTSLIKE
LAKSHKTEHKIDIKDLKYIAESIIEVLKKRFPEEFDEKAKEAVEKVLNLFIEKIEEFYKKGGGSGSHHWGSTHHHHHH

dnMb9

ATGTCAGGAAGCAAACTGACCGAAGAAGAATGGAAAACCGTGTTTAAAATTTTTGCGCTGGTGGAAAAAGATCTGGAAGGCTTTGGCCTGGCG
GTGCTGATTCGCACCTTTACCCGTTATCCAGAAACCTTGAAAAAATTTCCACGTTTCGCGCATCTGAAGACCGTGGAAGAATTGCGTGCGAGC
CCGCTGCTGCGCGAACATGGCGTGACCGTGCTGAAAGCGCTGACCAAAATTGCGGAAGAACTGAAGAAAGGCAAAACCGGCACCCTCAAAAAA
CTGGCGGAAAGCCATAGCAAAGTGCATAAAATTCCGATTAGCGATTTAGAACGCATTGCCGAAGCGATTATTGAAGTGCTGGAAGAACGCTTT
CCGGAAGAGTTTGATGAAAAAGCGAAAGAAGCGGTGAAGAAGTTTCTGGATCTGTTTATCGAAAAACATGCGGAATTTGTGAAAAAAGGCGGC
GGTTCCGGCAGCCATCATTGGGGCAGCACCCACCACCACCACCACCAC

MSGSKLTEEEWKTVFKIFALVEKDLEGFGLAVLIRTFTRYPETLKKFPRFAHLKTVEELRASPLLREHGVTVLKALTKIAEELKKGKTGTLKK
LAESHSKVHKIPISDLERIAEAIIEVLEERFPEEFDEKAKEAVKKFLDLFIEKHAEFVKKGGGSGSHHWGSTHHHHHH

dnMb10

ATGTCAGGAGATGCGGAAAAACAGGCGCTGGTGGCGAGCATTTTTGCGAAATTTGAAGCGGATCTGGAAGGCTTTGGCAAAGCGGTGCTGATT
AAAACCTTTACCAAACATCCGGAAACCCGCAAAAAATTTCCGCGCTTTAAACATCTGAAAAGCGTGGAAGAACTGGAAAAAAGCGAAGAACTG
AAAGAACATGGCGTGACCGTGCTGACCGCGCTGCGCGAGATTAGCCTGGGCGAAAATCAGGATAAAAAGATTAAAGATCTGGCGACCAGCCAT
AAAGAAAAGCATAAAATTCCGATTGAAGATTTGGAAGTGATTGCGGCGGCGATTTTAGAAGTGGCGAAGGAACGCTTTCCGGAAGAATTTGAT
GAGGCGGCGCAGGCAGCGCTGCAGGAATTTCTGGATGATTTTATTAGCAAATTAAAAGAATATTTTGAAGGCGGCGGTTCCGGCAGCCATCAT
TGGGGCAGCACCCACCACCACCACCACCAC

MSGDAEKQALVASIFAKFEADLEGFGKAVLIKTFTKHPETRKKFPRFKHLKSVEELEKSEELKEHGVTVLTALREISLGENQDKKIKDLATSH
KEKHKIPIEDLEVIAAAILEVAKERFPEEFDEAAQAALQEFLDDFISKLKEYFEGGGSGSHHWGSTHHHHHH

dnMb11

ATGTCAGGAAAACTGAGCGAAGAAGAAAAAGAAATTGTGCTGAAAATTTTTGCGCTGGTGGAAAAGGATCTGGAAGGCTTTGGCAAAGAAGTG
CTGATTAAAACCTTTCTGAAATATCCGGAAACCCTGAAAAAATTTCCGCGCTTTAAACATCTGAAAACCGAGGAAGAACTGAAAGCGTCGGAA
GAGTTGAAAGAACATGGCGTGACCGTGTTAAAAGCGCTGATTGAAATCTTTAAAAATGAAGATGAAGAAAAACTGAAGGAGCTGGCGAAAAGC
CATAAAGAAGAGCATAAAATTCCGATTGAAGATTTAGAGAAAATTGCGGAAGCGATTATTGAAGTACTGAAAGAGAAATACCCGGAAGAATTT
GATGAAGAAGCGGAGGAGGCGGTGAAAAAGTTTTTAAAACTGTTTATCGAGAAGCTCAAAGAATATCGCGAAGGCGGCGGTTCCGGCAGCCAT
CATTGGGGCAGCACCCACCACCACCACCACCAC

MSGKLSEEEKEIVLKIFALVEKDLEGFGKEVLIKTFLKYPETLKKFPRFKHLKTEEELKASEELKEHGVTVLKALIEIFKNEDEEKLKELAKS
HKEEHKIPIEDLEKIAEAIIEVLKEKYPEEFDEEAEEAVKKFLKLFIEKLKEYREGGGSGSHHWGSTHHHHHH

dnMb12

ATGTCAGGACTGAATCTGAGCCCGGAAGATAAAGCGAAAGTGCTGGAAATTTTTGCGCTGGTGGAAGAAGATCTGGAAGGCTTTGGCCGCGAA
GTTCTGATTCTGACCTTTACCAAACATCCGGAAACCCTGAAAAAATTTCCACGCTTTGCGCATCTGAAAACCGAAGAGGAACTGCGCGCGAGC
GAAGAACTGAAAGAACATGGCGTGACCGTGCTGAAAGCGCTGCGTGCGATTCTGGAAAAAGGCGATGAAGAGCTGTTGAAGAAACTGGCGGAA
AGCCATACCAAAGAACATAAAATTCCGGTGAGCGATTTGGAAGTGATTGCGGAAAGCATTATTGAAGTGGCGAAAAAACGCTTTCCGGAGGAA
TTTGGTGAAGAAGCGCAGGCGGCGCTGAAGAAGTTTTTAGAAGAATTTATCGAAAAATGGAAAGAATATCAGGAGGAGTTTAAAGGCGGCGGT
TCCGGCAGCCATCATTGGGGCAGCACCCACCACCACCACCACCAC

MSGLNLSPEDKAKVLEIFALVEEDLEGFGREVLILTFTKHPETLKKFPRFAHLKTEEELRASEELKEHGVTVLKALRAILEKGDEELLKKLAE
SHTKEHKIPVSDLEVIAESIIEVAKKRFPEEFGEEAQAALKKFLEEFIEKWKEYQEEFKGGGSGSHHWGSTHHHHHH

dnMb13

ATGTCAGGAGATGAACGCAATAAACTGGTGCTGAGCGCGTTTGCGCTGGTGCGCGAAGATCTGGAAGAAATTGGCGCGGAAGTGCTGATTCTG
ACCTTTACCGAAAATCCGGAAACCCTGAAAAAATTTCCGCGCTTTGCGCATCTGAAAACCGAAGAAGAACTGAAAAAAAGCCCGCTGCTGAAA
GAACATGGCGTGACCGTGCTGAATGCGCTGATTGAAATTGCGGAATTAAAATATAGCGGCGGCGATTATGAAAGCCTGGTGAAAGAGCTGGCG
AAAAGCCATAAAGAAAAACATAAAATTCCGATTGAAGATTTGAAAGCGATTGCAGAAGCCATCTTGAAAGTCTTAAAAAAACGCTATCCAGAA
GAATTTGGTGAGAAGACCCAGGCGGCGTTGAAGGAGTTTCTGGATGAATTTATTGAACTGACCGAGAAATATTATAAAGGCGGCGGTTCCGGC
AGCCATCATTGGGGCAGCACCCACCACCACCACCACCAC

MSGDERNKLVLSAFALVREDLEEIGAEVLILTFTENPETLKKFPRFAHLKTEEELKKSPLLKEHGVTVLNALIEIAELKYSGGDYESLVKELA
KSHKEKHKIPIEDLKAIAEAILKVLKKRYPEEFGEKTQAALKEFLDEFIELTEKYYKGGGSGSHHWGSTHHHHHH

dnMb14

ATGTCAGGAGAAAAAGAAAAAAATGAACTGGTGCTGAAAGCGTTTGAACTGATTGAAAAGGATCTGGAAGGCTTTGGCAGCGAAGTGCTGATT
CTGACCTTTACCAAACATCCGGAAACCCTGAAAAAATTTCCGCGCTTTAAACATCTGAAAACCGAAGAAGAATTTAAAGCGAGCGAAGAACTG
AAAGAACATGGCGTGACCGTGTTGAAGGCGCTGATCGAAATTGCGAAATTAAAAGTGAGCGGCGAAGATTATGATAGCCTGATTAAAGAGCTG
GCGAAAAGCCATAAAACCAAACATAAAATTCCGATTGAATATTTGAAATATATTGCGGATGCGATTTTGGAAGTGGCAAAAAAACGCTTTCCG
AAAGAGTTTGATGAGAAGACCTATGCGGCGTTAAAGGAATTTCTGGATTATTTTATTGAGAAAATTGAGAAATATTATAAAGGCGGCGGTTCC
GGCAGCCATCATTGGGGCAGCACCCACCACCACCACCACCAC

MSGEKEKNELVLKAFELIEKDLEGFGSEVLILTFTKHPETLKKFPRFKHLKTEEEFKASEELKEHGVTVLKALIEIAKLKVSGEDYDSLIKEL
AKSHKTKHKIPIEYLKYIADAILEVAKKRFPKEFDEKTYAALKEFLDYFIEKIEKYYKGGGSGSHHWGSTHHHHHH

dnMb15

ATGTCAGGAGAAGAAGAAGAAAAACAGATTGTGCTGGAACTGTTTGCGAAAGTGGAAGAGGATCTGGAAGGCATTGGCCTGGAAGTGCTGATT
CTGACCTTTACCAAACATCCAGAAACCCGTAAAAAATTTCCACGTTTTGCGCATCTGACCACCGAAGCGCAGCTGCAGGCGAGCCCGGAACTG
AAACAGCATGGCGTGACCGTGCTGAAAGCGCTGATTACCATTGCCAAACTGTATTATGAAGGCAAAGATTACGAAAGCCTGATTAAAGAACTG
GCGAAAAGCCATAAAGAGGAACATAAAATTCCGATTGAATATTTGGAATATATTAGCGAAAGCATTTTGGAGGTTCTGAAAAAACGCTTTCCG
GAATTTTTTGGTGAGAAAGCCCAGGCGGCGGTGCGCAAATTTCTGGATTTTTTTATTAGCAAATTGAAAGAATATTACGAAGGCGGCGGTTCC
GGCAGCCATCATTGGGGCAGCACCCACCACCACCACCACCAC

MSGEEEEKQIVLELFAKVEEDLEGIGLEVLILTFTKHPETRKKFPRFAHLTTEAQLQASPELKQHGVTVLKALITIAKLYYEGKDYESLIKEL
AKSHKEEHKIPIEYLEYISESILEVLKKRFPEFFGEKAQAAVRKFLDFFISKLKEYYEGGGSGSHHWGSTHHHHHH

dnMb16

ATGTCAGGAGCGGAAGAAGAAAAAGAAAAAGTGCTGAGCATTTTTAAACTGGTGGAAAAGGATAAAAAAACCATTGGCAGCGAAGTCCTGATT
ATTACCTTTACCAAAAATCCGGAAACCAAGAAGAAATTTCCGCGCTTTAAAGATCTGAAAACCGTGGAAGAACTGAAAGCGAGCGAAAAAGTG

AAAGATCATGGCGTGACCGTGCTGGATGCGCTGATTGAATGGGCGCGCCTGCATGTGGAAGGCAAAGATTATGATAGCCTGGTGAAAAAACTG
GCGGAAAGCCATAAGAAGGAACATAAAATTCCGATTGAAGATTTGAAAAGCATTGCGGACGCCTTGATCGAAGTTTTAAAGAAATTTTATCCA
GAGGAATTTGGCGAAGAAGCGCAGGCGGCGGTGCAGAAACTGCTGAATTATTTTATTGAGAAGTTGAAACAGTATTATGAAGGCGGCGGTTCC
GGCAGCCATCATTGGGGCAGCACCCACCACCACCACCACCAC

MSGAEEEKEKVLSIFKLVEKDKKTIGSEVLIITFTKNPETKKKFPRFKDLKTVEELKASEKVKDHGVTVLDALIEWARLHVEGKDYDSLVKKL
AESHKKEHKIPIEDLKSIADALIEVLKKFYPEEFGEEAQAAVQKLLNYFIEKLKQYYEGGGSGSHHWGSTHHHHHH

dnMb17

ATGTCAGGAATTAAACTGAGCGAAGAAGAAAAAAAACTGGTGCTGGAAATTTTTAAGCTGTTTGAAGAGAATCTGGAAGAATTTGGCAAAGAA
GTGCTGATTACCACCTTTACCAAACATCCGGAAACCAAAAAAAAATTTCCGCGCTTTGCGCATCTGAAAACCGAGGAGGAATTTCTGGCGAGC
CCGGAACTGGCGAAACATGGCGTGACCGTGCTGAATGCGCTGATTGAAATTGCGAAACTGTATTTAGAAGGCAAGGATTATCGCAGCCTGATT
AAAAAGCTGGCAAAAAGCCATAAACTGGAACATAAAATTCCGATTGAAGATTTGAAATATATTGCGGATGCGATTATTGAAGTGGCCAAAAAG
TTCTTTCCAGAAAAATTCGGTGAAAAAGCGCAGGAAGCGCTGAAGAAGTTCCTGAATTATTTTATTGAGGAGTTGGAAAAAGAATATGAAAAA
CTGGGCGGCGGTTCCGGCAGCCATCATTGGGGCAGCACCCACCACCACCACCACCAC

MSGIKLSEEEKKLVLEIFKLFEENLEEFGKEVLITTFTKHPETKKKFPRFAHLKTEEEFLASPELAKHGVTVLNALIEIAKLYLEGKDYRSLI
KKLAKSHKLEHKIPIEDLKYIADAIIEVAKKFFPEKFGEKAQEALKKFLNYFIEELEKEYEKLGGGSGSHHWGSTHHHHHH

dnMb18

ATGTCAGGAGATGAAGAAAAGAAAAAACTGGTGCTGGAAGCGTTTGAATTGGTGGAAAAAGATATTGAAGGCATTGGCGCGGAAGTGCTGAAA
CTGACCTTTGAAAAACATCCGGAAACCCTGGAGAAATTTCCGCGCCTGAAAGAATTACATGCGGCGGGCAGCCCGGAACTGGAAGAACTGTTG
AAGGAACATGGCGCGACCGTGTTGAAAGCGCTGATTGAAATTGCGCGCTTAAAAATTAGCGGCGGCGATTATCTGAGCCTGGTGAAAGAGCTG
GCGAAAAGCCATAAAGAAGAGCATAAAATTCCGATTGAAGATCTGAAGAAAATCGCCGAAGCCCTGCTGGAGGTTTTGAAGGAAAAATATCCA
GAAGAATTTGGCGAAGAAACCCAGGAGGCTCTGAAAGAATTTCTGGATTGGTTTATCGAGGAGCTGGAAAAGGAATTTAAGGAAGGCGGCGGT
TCCGGCAGCCATCATTGGGGCAGCACCCACCACCACCACCACCAC

MSGDEEKKKLVLEAFELVEKDIEGIGAEVLKLTFEKHPETLEKFPRLKELHAAGSPELEELLKEHGATVLKALIEIARLKISGGDYLSLVKEL
AKSHKEEHKIPIEDLKKIAEALLEVLKEKYPEEFGEETQEALKEFLDWFIEELEKEFKEGGGSGSHHWGSTHHHHHH

dnMb19

ATGTCAGGAAGCGAAGAAAAAGCGGCGTTGGTGCTGGCGCTGTTTGATCGCGTGGAAGCGGATCGCGAAGAAATTGGTGCGGCGGTGCTGCGC
CGCACCTTTGAAGAACATCCGGAAACCCTGAAAAAATTTCCGCGCTTTCTGGAACTGTATAAAAAAGGTAGCCCAGAACTGGATGCGCTGCTG
AAAGAGCATGGCAAAACCGTGCTGGACGCGCTGATTGAAATTGCGCGCCTGCGCTATAGCGGCGAAGATTATCGCAGCCTGATTAAAGAGCTG
GCGAAAAGCCATAAAGAAGAACATAAAATTCCAATTGAAGATCTGCGCCATATTGCGGAAGCGTTGTTGGCCGTTTTGGCGGAACGCTTTCCG
GATGAATTTGGTCCAGAAGCGCGCGCGGCACTGACCGATTTTTTGGATTGGTTTATCGCGGAAATTGAGGAGGAATATAAGAAAGGCGGCGGT
TCCGGCAGCCATCATTGGGGCAGCACCCACCACCACCACCACCAC

MSGSEEKAALVLALFDRVEADREEIGAAVLRRTFEEHPETLKKFPRFLELYKKGSPELDALLKEHGKTVLDALIEIARLRYSGEDYRSLIKEL
AKSHKEEHKIPIEDLRHIAEALLAVLAERFPDEFGPEARAALTDFLDWFIAEIEEEYKKGGGSGSHHWGSTHHHHHH

dnMb20

ATGTCAGGACTGAGCGAAGAAGAATGGAAAATTGTGCTGGAAATTTTTGCGTTAGTTCGTGAAGATTTAGCGGGTGTTGGTGCGGCGGTTTTA
GAACGTACCTTTGCGACCCATCCAGAAACCTTAAAAAAATTTCCACGTTTTCTCGCGGCAGCGGAAGCGGGCGTGTTGGATCGTGCGCTGCTG
GCCGCGCATGGCGAAACCGTGCTGACCGCGCTGATTGAAATTGCGGAAAGCAAACTGGATCCGGAACTGATTAAGAAACTGGCGGAAAGCCAT
GTGAAAGAACATAAAATTCCGATTGAATATCTGCGCGCGATTGCCGATAGCCTGATCGCGGTCCTGAAAGAACGCTATCCAGAGCGCTTTGGT
GAAAAAGCGCAGGAGGCGGTTAAAAAGTTTCTGGATCTGTTTATTGAAAAGTTTGAAGAAGAAGCGGAAAAAGAAAAAGGCGGCGGTTCCGGC
AGCCATCATTGGGGCAGCACCCACCACCACCACCACCAC

MSGLSEEEWKIVLEIFALVREDLAGVGAAVLERTFATHPETLKKFPRFLAAAEAGVLDRALLAAHGETVLTALIEIAESKLDPELIKKLAESH
VKEHKIPIEYLRAIADSLIAVLKERYPERFGEKAQEAVKKFLDLFIEKFEEEAEKEKGGGSGSHHWGSTHHHHHH

dnMb21

ATGTCAGGAAGCTTAACCCCAGAAGAATTAGCGATTGTGAAAGCGCTGTTTGCGCGCGTGCGCGAAGATCTGGAAGGCGTGGGCGCGGAAGTG
CTGCGCTTGACCTTTGAAAAACATCCAGAAACCTTAAAAAAATTTCCACGTTTTTTGGAATTGAAGAAAGCGGGCAGCCCGGAACTGGAAGCG
GAGCTGCGTGCGCATGGCGTGACCGTGCTGACCGCGCTGATTGAACTTGCGGATAATTATGAAGGCAATAATGAAACTCTGGAAAAACTGGCC
GAAAGCCATACCAAAGTGCATAAAATTCCGGTGAGCGATCTGAAGAATATTGCGGCGGCGATTATTGAAGTCCTGAAAGAACGCTTTCCGGAA
GAGTTTGGCGAAGAAGCGCAGGCGGCGTTTACCAAATTTTTAGATAAATTTATTAAAGATATTGCGGAGCTGCAGAAAAAATTTGAAGGCGGC
GGTTCCGGCAGCCATCATTGGGGCAGCACCCACCACCACCACCACCAC

MSGSLTPEELAIVKALFARVREDLEGVGAEVLRLTFEKHPETLKKFPRFLELKKAGSPELEAELRAHGVTVLTALIELADNYEGNNETLEKLA
ESHTKVHKIPVSDLKNIAAAIIEVLKERFPEEFGEEAQAAFTKFLDKFIKDIAELQKKFEGGGSGSHHWGSTHHHHHH

Native Mb, 3RGK

ATGTCAGGAGGCCTGAGCGATGGCGAATGGCAGCTGGTGCTGAACGTGTGGGGCAAAGTGGAAGCGGATATTCCGGGCCATGGCCAGGAAGTG
CTGATTCGCCTGTTTAAAGGTCATCCGGAAACCCTGGAAAAGTTCGACCGCTTTAAACATCTGAAATCTGAAGATGAGATGAAAGCGAGCGAA
GATCTGAAGAAACATGGCGCGACCGTGCTGACCGCGCTGGGCGGTATTCTGAAGAAGAAAGGCCATCACGAAGCCGAAATTAAACCGCTGGCG
CAGAGCCATGCGACCAAACATAAAATTCCGGTGAAGTACCTGGAATTTATCAGCGAAGCGATTATTCAGGTGCTGCAGAGCAAACATCCGGGC
GATTTTGGCGCGGATGCGCAGGGTGCGATGAACAAAGCGCTGGAACTGTTTCGCAAAGATATGGCGAGCAACTATAAAGAACTGGGTTCCGGC
AGCCATCATTGGGGCAGCACCCACCACCACCACCACCAC

MSGGLSDGEWQLVLNVWGKVEADIPGHGQEVLIRLFKGHPETLEKFDRFKHLKSEDEMKASEDLKKHGATVLTALGGILKKKGHHEAEIKPLA
QSHATKHKIPVKYLEFISEAIIQVLQSKHPGDFGADAQGAMNKALELFRKDMASNYKELGSGSHHWGSTHHHHHH

TEV sequences

N-terminal tag + linker and C-terminal linker are highlighted in green.

hyperTEV56

ATGAGCCACCACCACCACCACCACTCAGGAATGGAAAGCGCGGCGCCGGGCCCGCGCGATTATAACCCGATCAGCGATACCATTGTGAAACTG
ACCAACACCTCTGATGGCTATAGCATTAGCCTGTATGGCATTGGCTTTGGCCCGCTGATCATTACCAACGCGCATTTATTTCGCCGCAACAAC
GGCACCCTGACCGTGACCAGCAAACATGGCACCTTCACCATTGAAAACACCACCACCCTGCAGCTGCATCTGATTGAAGGCCGCGATCTGGTG
ATTATTAAAATGCCGAAAGATTTTCCGCCGTTTCCGACCGATCTGGTGTTTCGCGAACCGGTGGAAGGCGAGAAAATTACCCTGGTGACCCGC
AACTTTCAGACCAAAGAACCGACCAGCGAAGTGAGCGATGTGAGCAGCACCTATCCGAGCAGCGATGGCGTGTTTTGGAAACATTGGATTCCG
ACCAAAGATGGTCAGTGCGGCAGCCCGATGGTGAGCGTGGAAGATGGCAGCATTGTGGGCATTCATAGCGCGAGCAACTTTACCAATACCAAC
AACTATTTTACCGCGGTGCCGCCGGATTTCATGGATCTGCTGACCAACGATAGCCTGCAGAAATGGATTAGCGGCTGGAGCCTGAACAGCGAT
AGCGTTGAATGGGGCGGCCATAAAGTGTTTATGGATAAACCGGGTTCC

MSHHHHHHSGMESAAPGPRDYNPISDTIVKLTNTSDGYSISLYGIGFGPLIITNAHLFRRNNGTLTVTSKHGTFTIENTTTLQLHLIEGRDLV
IIKMPKDFPPFPTDLVFREPVEGEKITLVTRNFQTKEPTSEVSDVSSTYPSSDGVFWKHWIPTKDGQCGSPMVSVEDGSIVGIHSASNFTNTN
NYFTAVPPDFMDLLTNDSLQKWISGWSLNSDSVEWGGHKVFMDKPGS

hyperTEV60

ATGAGCCACCACCACCACCACCACTCAGGAGCGGAAAGCGCGGCGCCGGGCCCGCGCGATTATAACCCGATTAGCGATACCATTGTTCTGCTG
ACCAATACCAGCGATGGCTATAGCATTAGCCTGTATGGCATTGGCTTTGGCCCGCTGATTATTACCAACGCGCACCTGTTTCGCCGCAACAAC
GGCACCCTGACCATTACCAGCAAACATGGCACCTTTACCATTAGCAACACCACCACCCTGAAACTGCATCTGATCGAAGGCCGCGATCTGGTG
CTGATTGAAATGCCGAAAGATTTTCCGCCGTTTCCGACCAACCTGGTGTTTCGTGAACCGGTGGTGGGCGAAGAAATTGTGCTGGTGACCCGC
AACTTTCAGACCAAAACCCCGACCAGCGAAGTGAGCGATGTGAGCACCACCTATCCGAGCTCCGATGGCGTGTTTTGGAAACATTGGATTCCG
ACGAAAGATGGCCAGTGCGGCAGCCCGATGGTGAGCGTGACCGATGGCAGCATTGTGGGCATTCATAGCGCGAGCAACTTTACCAACACCAAC
AACTATTTTACCGCGGTGCCGCCGGATTTTATGCGCCTGCTGACCGATCCGAGCCTGCAGAAATGGGTGAGCGGCTGGAGCCTGAACAGCGAT
AGCGTGGAATGGGGCGGCCATAAAGTGTTTATGGATAAACCGGGTTCC

MSHHHHHHSGAESAAPGPRDYNPISDTIVLLTNTSDGYSISLYGIGFGPLIITNAHLFRRNNGTLTITSKHGTFTISNTTTLKLHLIEGRDLV
LIEMPKDFPPFPTNLVFREPVVGEEIVLVTRNFQTKTPTSEVSDVSTTYPSSDGVFWKHWIPTKDGQCGSPMVSVTDGSIVGIHSASNFTNTN
NYFTAVPPDFMRLLTDPSLQKWVSGWSLNSDSVEWGGHKVFMDKPGS

hyperTEV89

ATGAGCCACCACCACCACCACCACTCAGGAGCGGAAAGCGCGGCGCCGGGCCCGCGCGATTATAACCCGATTAGCAGCACCATTGTGCGCCTG
ACCAACACCAGCGACGGCCATAGCATTAGCCTGTTTGGCATTGGCTTTGGCCCGCTGATTATTACCAACGCGCATTTATTTCGCCGCAACAAC
GGCACCCTGACCATTACCAGCCTGCATGGCACCTTTACCATTAGCAACACCACCACCCTGAAACTGCATCTGATTGAAGGCCGCGATCTGGTG
ATTATCAAAATGCCGAAAGATTTTCCGCCGTTTCCGACCACCCTGGAATTTCGCGAACCGGTGGTGGGCGAAGATATTGTGCTGGTGACCCGC
AACTTTCAGGATAAAGATCCGACCAGCGAAGTGAGCGATACCAGCACCACCGAACCGAGCAGTGATGGCGTGTTTTGGAAACATTGGATCCCG
ACCAAAGATGGCCAGTGCGGCAGCCCGATGGTGAGCGTTAGCGATGGCAGCATTGTGGGCATTCATAGCGCGAGCAACTTCACCAATACCAAC

AACTATTTTACCGCGGTGCCGCCGAACTTTATGGATCTGCTGACCGATCCGAGCCTGCAGAAATGGATTAGCGGCTGGAGCCTGAACGCGGAT
AGCGTGGATTGGGGCGGCCATAAAGTGTTCATGGATAAACCGGGTTCC

MSHHHHHHSGAESAAPGPRDYNPISSTIVRLTNTSDGHSISLFGIGFGPLIITNAHLFRRNNGTLTITSLHGTFTISNTTTLKLHLIEGRDLV
IIKMPKDFPPFPTTLEFREPVVGEDIVLVTRNFQDKDPTSEVSDTSTTEPSSDGVFWKHWIPTKDGQCGSPMVSVSDGSIVGIHSASNFTNTN
NYFTAVPPNFMDLLTDPSLQKWISGWSLNADSVDWGGHKVFMDKPGS

TEVd (PDB: 1LVM)[2]

ATGAGCCACCACCACCACCACCACTCAGGAGGCGAAAGCCTGTTCAAAGGCCCGCGCGATTATAACCCGATTAGCAGCACCATTTGCCATCTG
ACCAACGAAAGCGATGGCCATACCACCAGCCTGTATGGCATTGGCTTTGGCCCGTTTATCATTACCAACAAACATTTATTTCGCCGCAACAAC
GGCACCCTGCTGGTGCAGAGCCTGCATGGCGTGTTTAAAGTGAAGAATACCACGACCCTGCAGCAGCATCTGATTGATGGCCGCGATATGATT
ATTATTCGCATGCCGAAAGATTTTCCGCCGTTTCCGCAGAAACTGAAATTTCGCGAACCGCAGCGTGAAGAACGCATTTGTCTGGTGACCACC
AACTTTCAGACGAAAAGCATGAGCAGCATGGTGAGCGATACCAGCTGCACCTTTCCGAGCAGCGATGGTATCTTTTGGAAACATTGGATTCAG
ACCAAAGATGGTCAGTGCGGCAGCCCGCTGGTGAGCACCCGTGATGGCTTTATTGTGGGCATTCATAGCGCGAGCAACTTTACCAATACCAAT
AACTATTTTACCAGCGTGCCGAAGAACTTTATGGAACTGCTGACCAACCAGGAAGCGCAGCAGTGGGTGAGCGGCTGGCGCCTGAACGCGGAT
AGCGTGCTGTGGGGCGGCCATAAAGTGTTTATGGATAAACCGGGTTCC

MSHHHHHHSGGESLFKGPRDYNPISSTICHLTNESDGHTTSLYGIGFGPFIITNKHLFRRNNGTLLVQSLHGVFKVKNTTTLQQHLIDGRDMI
IIRMPKDFPPFPQKLKFREPQREERICLVTTNFQTKSMSSMVSDTSCTFPSSDGIFWKHWIQTKDGQCGSPLVSTRDGFIVGIHSASNFTNTN
NYFTSVPKNFMELLTNQEAQQWVSGWRLNADSVLWGGHKVFMDKPGS

S219V[2]

ATGAGCCACCACCACCACCACCACTCAGGAGGCGAAAGCCTGTTCAAAGGCCCGCGCGATTATAACCCGATTAGCAGCACCATTTGCCATCTG
ACCAATGAAAGCGATGGCCATACCACCAGCCTGTATGGCATTGGCTTTGGCCCGTTTATTATTACCAACAAACATTTATTTCGCCGCAACAAC
GGCACCCTGCTGGTGCAGAGCCTGCATGGCGTGTTTAAAGTTAAGAACACCACCACCCTGCAGCAGCATCTGATTGATGGCCGCGATATGATC
ATTATTCGCATGCCGAAAGATTTTCCGCCGTTTCCGCAGAAACTGAAATTTCGCGAACCGCAGCGCGAAGAACGCATTTGCCTGGTGACCACC
AACTTTCAGACCAAAAGCATGAGCAGCATGGTGAGCGATACCAGCTGCACCTTTCCGAGCAGCGATGGTATCTTTTGGAAACATTGGATTCAG
ACGAAAGATGGCCAGTGCGGCAGCCCGCTGGTGAGCACCCGTGATGGCTTTATTGTGGGCATTCATAGCGCGAGCAACTTTACCAACACCAAC
AACTATTTTACCAGCGTGCCGAAGAATTTTATGGAACTGCTGACCAACCAGGAAGCGCAGCAGTGGGTGAGCGGCTGGCGCCTGAACGCGGAT
AGCGTGCTGTGGGGCGGCCATAAAGTGTTTATGGTGAAACCGGAAGAACCGTTTCAGCCGGTGAAAGAAGCGACCCAGCTGATGAACGAAGGT
TCC

MSHHHHHHSGGESLFKGPRDYNPISSTICHLTNESDGHTTSLYGIGFGPFIITNKHLFRRNNGTLLVQSLHGVFKVKNTTTLQQHLIDGRDMI
IIRMPKDFPPFPQKLKFREPQREERICLVTTNFQTKSMSSMVSDTSCTFPSSDGIFWKHWIQTKDGQCGSPLVSTRDGFIVGIHSASNFTNTN
NYFTSVPKNFMELLTNQEAQQWVSGWRLNADSVLWGGHKVFMVKPEEPFQPVKEATQLMNEGS

TEV1Δ[27]

ATGAGCCACCACCACCACCACCACTCAGGAGGCGAAAGCCTGTTTAAAGGCCCGCGCGATTATAACCCGATTAGCAGCACCATTTGCCATCTG
ACCAACGAAAGCGATGGCCATACCACCAGCCTGTATGGCATTGGCTTTGGCCCGTTTATTATTACCAATAAACATTTATTTCGCCGCAACAAC
GGCACCCTGCTGGTGCAGAGCCTGCATGGCGTGTTTAAAGTGAAGAATACCACGACCCTGCAGCAGCATCTGATTGATGGCCGCGATATGATT
ATTATTCGCATGCCGAAAGATTTTCCGCCGTTTCCGCAGAAACTGAAATTTCGCGAACCGCAGCGCGAAGAACGCATCTGCCTGGTGACCACC
AACTTTCAGACCAAAAGCATGAGCAGCATGGTGAGCGATACCAGCTGCACCTTTCCGAGCAGCGACGGCATTTTCTGGAAACATTGGATTCAG
ACGAAAGATGGTCAGTGCGGCAACCCGCTGGTGAGCACCCGCGATGGCTTTATTGTGGGCATTCATAGCGCGAGCAACTTTACCAACACCAAC
AACTATTTTACCAGCGTGCCGAAGAACTTTATGGAACTGCTGACCAACCAGGAAGCGCAGCAGTGGGTGAGCGGCTGGCGCCTGAACGCGGAT
AGCGTGCTGTGGGGCGGCCATAAAGTGTTTATGGTGGGTTCC

MSHHHHHHSGGESLFKGPRDYNPISSTICHLTNESDGHTTSLYGIGFGPFIITNKHLFRRNNGTLLVQSLHGVFKVKNTTTLQQHLIDGRDMI
IIRMPKDFPPFPQKLKFREPQREERICLVTTNFQTKSMSSMVSDTSCTFPSSDGIFWKHWIQTKDGQCGNPLVSTRDGFIVGIHSASNFTNTN
NYFTSVPKNFMELLTNQEAQQWVSGWRLNADSVLWGGHKVFMVGS

superTEV[28]

ATGAGCCACCACCACCACCACCACTCAGGACCGCGCGATTATAACCCGATTAGCAGCACCATTGTGCATCTGACCAACGAAAGCGATGGCCAT
ACCACCAGCCTGTATGGCATTGGCTTTGGCCCGTTTATTATTACCAACAAACATTTATTTCGCCGCAACAACGGCACCCTGCTGGTGCAGAGC
CTGCATGGCGTGTTTAAAGTTAAGAACACCACCACCCTGCAGCAGCATCTGATTGATGGCCGCGATATGATTATTATCCGCATGCCGAAAGAT
TTTCCGCCGTTTCCGCAGAAACTGAAATTTCGCGAACCGCAGCGTGAAGAACGTATTGTGCTGGTGACCACCAACTTTCAGACCAAAAGCATG
AGCAGCATGGTGAGCGATACCAGCAGCACCTTTCCGAGCAGCGATGGTATTTTCTGGAAACATTGGATCCAGACCAAAGATGGCCAGTGCGGC

AGCCCGCTGGTGAGCACCCGTGATGGCTTTATTGTGGGCATTCATAGCGCGAGCAACTTTACCAACACCAATAACTATTTTACCAGCGTGCCG
AAGAACTTTATGGAACTGCTGACCAATCAGGAAGCGCAGCAGTGGGTGAGCGGCTGGCGCCTGAACGCGGATAGCGTGCTGTGGGGCGGCCAT
AAAGTGTTTATGGATAAACCGGGTTCC

MSHHHHHHSGPRDYNPISSTIVHLTNESDGHTTSLYGIGFGPFIITNKHLFRRNNGTLLVQSLHGVFKVKNTTTLQQHLIDGRDMIIIRMPKD
FPPFPQKLKFREPQREERIVLVTTNFQTKSMSSMVSDTSSTFPSSDGIFWKHWIQTKDGQCGSPLVSTRDGFIVGIHSASNFTNTNNYFTSVP
KNFMELLTNQEAQQWVSGWRLNADSVLWGGHKVFMDKPGS

MBP-TEVcs-FKBP-EGFP substrate

TEVcs is highlighted in orange, FKBP is highlighted in green, and EGFP is highlighted in yellow.

MKIEEGKLVIWINGDKGYNGLAEVGKKFEKDTGIKVTVEHPDKLEEKFPQVAATGDGPDIIFWAHDRFGGYAQSGLLAEITPDKAFQDKLYPF
TWDAVRYNGKLIAYPIAVEALSLIYNKDLLPNPPKTWEEIPALDKELKAKGKSALMFNLQEPYFTWPLIAADGGYAFKYENGKYDIKDVGVDN
AGAKAGLTFLVDLIKNKHMNADTDYSIAEAAFNKGETAMTINGPWAWSNIDTSKVNYGVTVLPTFKGQPSKPFVGVLSAGINAASPNKELAKE
FLENYLLTDEGLEAVNKDKPLGAVALKSYEEELAKDPRIAATMENAQKGEIMPNIPQMSAFWYAVRTAVINAASGRQTVDEALKDAQTNSSSN
NNNNNNNNNLGIEGRISTSGSGGGGGSMSENLYFQGSMGVQVETISPGDGRTFPKRGQTCVVHYTGMLEDGKKFDSSRDRNKPFKFMLGKQEV
IRGWEEGVAQMSVGQRAKLTISPDYAYGATGHPGIIPPHATLVFDVELLKLNEGGSGGSGGSGGSMVSKGEELFTGVVPILVELDGDVNGHKF
SVSGEGEGDATYGKLTLKFICTTGKLPVPWPTLVTTLTYGVQCFSRYPDHMKQHDFFKSAMPEGYVQERTIFFKDDGNYKTRAEVKFEGDTLV
NRIELKGIDFKEDGNILGHKLEYNYNSHNVYIMADKQKNGIKVNFKIRHNIEDGSVQLADHYQQNTPIGDGPVLLPDNHYLSTQSALSKDPNE
KRDHMVLLEFVTAAGITLGMDELYKSGRHHHHHH

REFERENCES

(1) Hubbard, S. R.; Hendrickson, W. A.; Lambright, D. G.; Boxer, S. G. X-Ray Crystal Structure of a Recombinant Human Myoglobin Mutant at 2·8 Å Resolution. *J. Mol. Biol.* **1990**, *213* (2), 215–218.

(2) Phan, J.; Zdanov, A.; Evdokimov, A. G.; Tropea, J. E.; Peters, H. K., 3rd; Kapust, R. B.; Li, M.; Wlodawer, A.; Waugh, D. S. Structural Basis for the Substrate Specificity of Tobacco Etch Virus Protease. *J. Biol. Chem.* **2002**, *277* (52), 50564–50572.

(3) Remmert, M.; Biegert, A.; Hauser, A.; Söding, J. HHblits: Lightning-Fast Iterative Protein Sequence Searching by HMM-HMM Alignment. *Nat. Methods* **2011**, *9* (2), 173–175.

(4) Wicky, B. I. M.; Milles, L. F.; Courbet, A.; Ragotte, R. J.; Dauparas, J.; Kinfu, E.; Tipps, S.; Kibler, R. D.; Baek, M.; DiMaio, F.; Li, X.; Carter, L.; Kang, A.; Nguyen, H.; Bera, A. K.; Baker, D. Hallucinating Symmetric Protein Assemblies. *Science* **2022**, *378* (6615), 56–61.

(5) Dang, B.; Mravic, M.; Hu, H.; Schmidt, N.; Mensa, B.; DeGrado, W. F. SNAC-Tag for Sequence-Specific Chemical Protein Cleavage. *Nat. Methods* **2019**, *16* (4), 319–322.

(6) Edward A. Berry, B. L. T. Simultaneous Determination of Hemes A, B, and c from Pyridine Hemochrome Spectra. *Analytical Biochemistry* **1987**, *161* (1), 1–15.

(7) Antonini, E. Hemoglobin and Myoglobin in Their Reactions with Ligands. *Front. Biol.* **1971**, *21*, 27–31.

(8) Jumper, J.; Evans, R.; Pritzel, A.; Green, T.; Figurnov, M.; Ronneberger, O.; Tunyasuvunakool, K.; Bates, R.; Žídek, A.; Potapenko, A.; Bridgland, A.; Meyer, C.; Kohl, S. A. A.; Ballard, A. J.; Cowie, A.; Romera-Paredes, B.; Nikolov, S.; Jain, R.; Adler, J.; Back, T.; Petersen, S.; Reiman, D.; Clancy, E.; Zielinski, M.; Steinegger, M.; Pacholska, M.; Berghammer, T.; Bodenstein, S.; Silver, D.; Vinyals, O.; Senior, A. W.; Kavukcuoglu, K.; Kohli, P.; Hassabis, D. Highly Accurate Protein Structure Prediction with AlphaFold. *Nature* **2021**, *596* (7873), 583–589.

(9) Case, D. A.; Belfon, K.; Ben-Shalom, I. Y.; Brozell, S. R. Amber 2020: University of California. *San Franc.*

(10) Wang, J.; Wolf, R. M.; Caldwell, J. W.; Kollman, P. A.; Case, D. A. Development and Testing of a General Amber Force Field. *J. Comput. Chem.* **2004**, *25* (9), 1157–1174.

(11) Bayly, C. I.; Cieplak, P.; Cornell, W.; Kollman, P. A. A Well-Behaved Electrostatic Potential Based Method Using Charge Restraints for Deriving Atomic Charges: The RESP Model. *J. Phys. Chem.* **1993**, *97* (40), 10269–10280.

(12) Frish, M. J.; Trucks, J. W.; Schlegel, H. B.; Scuseria, G. E. Gaussian 16, Revision C. 01; Gaussian. *Inc.: Wallingford, CT, USA.*

(13) Jorgensen, W. L.; Chandrasekhar, J.; Madura, J. D.; Impey, R. W.; Klein, M. L. Comparison of Simple Potential Functions for Simulating Liquid Water. *J. Chem. Phys.* **1983**, *79* (2), 926–935.

(14) Andersen, H. C. Molecular Dynamics Simulations at Constant Pressure And/or Temperature. *J. Chem. Phys.* **1980**.

(15) Andrea, T. A.; Swope, W. C.; Andersen, H. C. The Role of Long Ranged Forces in Determining the Structure and Properties of Liquid Water. *J. Chem. Phys.* **1983**, *79* (9), 4576–4584.

(16) Miyamoto, S.; Kollman, P. A. Settle: An Analytical Version of the SHAKE and RATTLE Algorithm for Rigid Water Models. *J. Comput. Chem.* **1992**, *13* (8), 952–962.

(17) Darden, T.; York, D.; Pedersen, L. Particle Mesh Ewald: An N·log(N) Method for Ewald Sums in Large Systems. *J. Chem. Phys.* **1993**, *98* (12), 10089–10092.

(18) Sievers, F.; Wilm, A.; Dineen, D.; Gibson, T. J.; Karplus, K.; Li, W.; Lopez, R.; McWilliam, H.; Remmert, M.; Söding, J.; Thompson, J. D.; Higgins, D. G. Fast, Scalable Generation of High-Quality Protein Multiple Sequence Alignments Using Clustal Omega. *Mol. Syst. Biol.* **2011**, *7* (1), 539.

(19) Wang, J.; Lisanza, S.; Juergens, D.; Tischer, D.; Watson, J. L.; Castro, K. M.; Ragotte, R.; Saragovi, A.; Milles, L. F.; Baek, M.; Anishchenko, I.; Yang, W.; Hicks, D. R.; Expòsit, M.; Schlichthaerle, T.; Chun, J.-H.; Dauparas, J.; Bennett, N.; Wicky, B. I. M.; Muenks, A.; DiMaio, F.; Correia, B.; Ovchinnikov, S.; Baker, D. Scaffolding Protein Functional Sites Using Deep Learning. *Science* **2022**, *377* (6604), 387–394.

(20) Dauparas, J.; Anishchenko, I.; Bennett, N.; Bai, H.; Ragotte, R. J.; Milles, L. F.; Wicky, B. I. M.; Courbet, A.; de Haas, R. J.; Bethel, N.; Leung, P. J. Y.; Huddy, T. F.; Pellock, S.; Tischer, D.; Chan, F.; Koepnick, B.; Nguyen, H.; Kang, A.; Sankaran, B.; Bera, A. K.; King, N. P.; Baker, D. Robust Deep Learning-Based Protein Sequence Design Using ProteinMPNN. *Science* **2022**, *378* (6615), 49–56.

(21) Kabsch, W. XDS. *Acta Crystallogr. D Biol. Crystallogr.* **2010**, *66* (Pt 2), 125–132.

(22) Winn, M. D.; Ballard, C. C.; Cowtan, K. D.; Dodson, E. J.; Emsley, P.; Evans, P. R.; Keegan, R. M.; Krissinel, E. B.; Leslie, A. G. W.; McCoy, A.; McNicholas, S. J.; Murshudov, G. N.; Pannu, N. S.; Potterton, E. A.; Powell, H. R.; Read, R. J.; Vagin, A.; Wilson, K. S. Overview of the CCP4 Suite and Current Developments. *Acta Crystallogr. D Biol. Crystallogr.* **2011**, *67* (Pt 4), 235–242.

(23) McCoy, A. J.; Grosse-Kunstleve, R. W.; Adams, P. D.; Winn, M. D.; Storoni, L. C.; Read, R. J. Phaser Crystallographic Software. *J. Appl. Crystallogr.* **2007**, *40* (Pt 4), 658–674.

(24) Adams, P. D.; Afonine, P. V.; Bunkóczi, G.; Chen, V. B.; Davis, I. W.; Echols, N.; Headd, J. J.; Hung, L.-W.; Kapral, G. J.; Grosse-Kunstleve, R. W.; McCoy, A. J.; Moriarty, N. W.; Oeffner, R.; Read, R. J.; Richardson, D. C.; Richardson, J. S.; Terwilliger, T. C.;

Zwart, P. H. PHENIX: A Comprehensive Python-Based System for Macromolecular Structure Solution. *Acta Crystallogr. D Biol. Crystallogr.* **2010**, *66* (Pt 2), 213–221.

(25) Emsley, P.; Cowtan, K. Coot: Model-Building Tools for Molecular Graphics. *Acta Crystallogr. D Biol. Crystallogr.* **2004**, *60* (Pt 12 Pt 1), 2126–2132.

(26) Williams, C. J.; Headd, J. J.; Moriarty, N. W.; Prisant, M. G.; Videau, L. L.; Deis, L. N.; Verma, V.; Keedy, D. A.; Hintze, B. J.; Chen, V. B.; Jain, S.; Lewis, S. M.; Arendall, W. B., 3rd; Snoeyink, J.; Adams, P. D.; Lovell, S. C.; Richardson, J. S.; Richardson, D. C. MolProbity: More and Better Reference Data for Improved All-Atom Structure Validation. *Protein Sci.* **2018**, *27* (1), 293–315.

(27) Sanchez, M. I.; Ting, A. Y. Directed Evolution Improves the Catalytic Efficiency of TEV Protease. *Nat. Methods* **2020**, *17* (2), 167–174.

(28) Correnti, C. E.; Gewe, M. M.; Mehlin, C.; Bandaranayake, A. D.; Johnsen, W. A.; Rupert, P. B.; Brusniak, M.-Y.; Clarke, M.; Burke, S. E.; De Van Der Schueren, W.; Pilat, K.; Turnbaugh, S. M.; May, D.; Watson, A.; Chan, M. K.; Bahl, C. D.; Olson, J. M.; Strong, R. K. Screening, Large-Scale Production and Structure-Based Classification of Cystine-Dense Peptides. *Nat. Struct. Mol. Biol.* **2018**, *25* (3), 270–278.
